# Circular RNA cancer vaccines drive immunity in hard-to-treat malignancies

**DOI:** 10.7150/thno.77350

**Published:** 2022-08-29

**Authors:** Hongjian Li, Kun Peng, Kai Yang, Wenbo Ma, Shaolong Qi, Xinyang Yu, Jia He, Xin Lin, Guocan Yu

**Affiliations:** 1 Institute for Immunology and School of Medicine, Tsinghua University, Beijing 100084, China.; 2 Key Laboratory of Bioorganic Phosphorus Chemistry & Chemical Biology, Department of Chemistry, Tsinghua University, Beijing, 100084, China.; 3 School of Pharmaceutical Sciences, Tsinghua University, Beijing 100084, China.; 4 Tsinghua-Peking Center for Life Sciences, Beijing 100084, China.

**Keywords:** circular RNA, cancer vaccines, lipid nanoparticles, hard-to-treat malignancies, tumor immunotherapy

## Abstract

**Rationale:** Messenger RNA (mRNA) vaccine outperforms other kinds of cancer immunotherapy due to its high response rates, easy preparation, and wide applicability, which is considered as one of the most promising forms of next-generation cancer therapies. However, the inherent instability and insufficient protein expression duration of mRNA limit the efficacy and widespread application of the vaccine.

**Methods:** Here, we first tested the possibility of a novel circular RNA (circRNA) platform for protein expression and compare its duration with linear RNA. Then, we developed a lipid nanoparticle (LNP) system for circRNA delivery *in vitro* and *in vivo*. Next, the innate and adaptive immune response of circRNA-LNP complex was evaluated *in vivo*. The anti-tumor efficacy of circRNA-LNP was further confirmed in three tumor models. Finally, the possibility of combination therapy with circRNA-LNP and adoptive cell transfer therapy was further investigated in a late-stage tumor model.

**Results:** We successfully increased the stability of the RNA vaccine by circularizing the linear RNA molecules to form highly stable circRNA molecules which exhibited durable protein expression ability. By encapsulating the antigen-coding circRNA in LNP enabling *in vivo* expression, we established a novel circRNA vaccine platform, which was capable of triggering robust innate and adaptive immune activation and showed superior anti-tumor efficacy in multiple mouse tumor models.

**Conclusions:** Overall, our circRNA vaccine platform provides a novel prospect for the development of cancer RNA vaccines in a wide range of hard-to-treat malignancies.

## Introduction

Cancer immunotherapy represented by immune checkpoint blockade and adoptive cell transfer (ACT) therapy shed light on the possibility of overcoming this life-threatening disease 1-5. These therapies have already shown promising outcomes in hematological malignancies 6-8. However, attempts to use them in “immune-desert” and “immune-excluded” solid tumors remains unsatisfactory results, mainly due to the immunosuppressive tumor microenvironment 9-11. More powerful means of immunotherapy are under pressing request to treat various malignancy patients. Among all kinds of clinically tested cancer immune therapies, cancer vaccine is the one that shows the most promising application potentials 12-17.

Cancer vaccine induces anti-tumor responses by expressing tumor antigens *in vivo* which allows antigen-presenting cells presentation and activation of tumor antigen-specific T cells 18, 19. Among all the cancer vaccine subtypes, RNA-based cancer vaccine is considered as the most promising type, which is capable of providing rapid antigen expression in cytoplasm for a robust immune activation, avoiding the risk of genome integration or T cell tolerance 12-14, 20. RNA-based cancer vaccine is considered as the most brilliant option, which has proved its unparalleled superiority in various preclinical and clinical trials 20-22. To further optimize the efficacy of RNA vaccines, major efforts have been focused on increasing the stability of RNA molecules that is inherently unstable and susceptible to wide existed RNase degradation. Previous strategies to increase the stability of mRNA molecules include the optimization of untranslated regions such as 5' and 3' translation regulating sequences, incorporation of a methylguanosine cap analog to protect mRNA from decapping enzymes, nucleoside modifications, and codon optimization 23-25. These strategies dramatically increase the cost of manufacturing RNA vaccines, while only yield modest improvements in RNA stability. Thus, alternative approaches to improve the stability of RNA molecules are desirable for fully unleashing the potential of RNA vaccines in cancer treatment.

Generally, linear mRNA molecule is sensitive to RNase degradation due to its linear conformation, which provides free nucleoside ends for RNase digestion. However, circular RNA (circRNA), a novel kind of RNA molecule, shows high stability and RNase resistance mainly contributes to its circular conformation 26-28. The naturally generated circRNA molecules are produced via a noncanonical splicing event called back-splicing, and most of them are considered splicing by-products 29. Most of these circRNA molecules are short and incapable of initiating protein translation due to a lack of cap-dependent translation initiation structures, and only functionalize as microRNA sponges and transcription regulators 29. Recent studies indicate that certain circRNAs can be translated into proteins with biological functions through cap-independent mechanisms 30. By incorporation of a cap-independent translation initiation structure (IRES) into the circRNA sequence, protein-coding circRNA has been generated that can initiate protein expression in eukaryotic cells 31, 32. Indeed, a circRNA vaccine against SARS-CoV-2 has been developed, which exhibits superb performance 33. Yet, circRNA-based cancer vaccine has not been developed and it is worth trying to evaluate the applicability of circRNA vaccine in tumor immunotherapy for optimization of the vaccine stability.

Herein, we verify the stability and the duration of protein expression initiated by circRNA and report the first trial to evaluate the efficacy of circRNA-based cancer vaccines in hard-to-treat malignancies. We employ lipid nanoparticles (LNP) to efficiently package and deliver circRNA molecules, promoting endosome escape and achieving robust circRNA translation *in vivo*. CircRNA-LNP drive suitable innate immune response and robust antigen specific cytotoxic T cell activation, which eliminate hard-to-treat tumors in mouse models. Intriguingly, the vaccination system can also combine with adoptive cell transfer therapies to exert a supercharged antitumor efficacy in a late-stage mouse tumor model. These results provide a proof-of-concept demonstration of the circRNA-LNP vaccine efficacy in cancer treatments. In terms of clinical translation, circRNA-LNP vaccine exhibits great potential to be used as both primary and adjuvant therapies for various malignancies.

## Results

### *In vitro* synthesis of translatable circRNA

**Figure 1:**
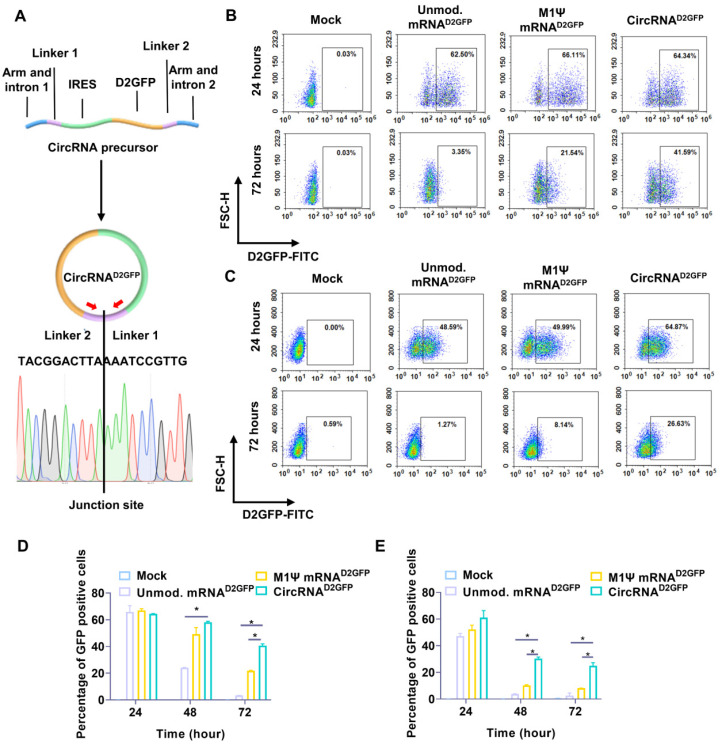
** Protein expression initiated by circRNA and linear mRNA. (A)** Upper panel, a scheme of the elements needed for circRNA^D2GFP^ production via the PIE system. Lower panel, sanger sequencing chromatograph for the junction site of the reverse-transcribed circRNA^D2GFP^ sample. Protein expression level of circRNA^D2GFP^ and the D2GFP-coding linear mRNA (mRNA^D2GFP^) in **(B)** HEK293T cells and **(C)** NIH3T3 cells. Statistical analysis of the percentage of GFP positive (**D**) HEK293T cells and (**E**) NIH3T3 cells at different time points. *p < 0.05.

We utilized a permuted intron-exon (PIE) system for the manufacturing of circRNA via a pair of intron and exon elements 31. A pair of homology arms and linkers were integrated at the indicated position of the circRNA backbone 31, and IRES element was used for translation initiation (Figure 1A). We employed destabilized GFP (D2GFP) with a half-life of approximately two hours as a model to identify the stability and persistence of circRNA *in vitro*. A D2GFP-coding circRNA (circRNA^D2GFP^) was synthesized, in which the coding sequence of D2GFP was inserted after the IRES element (Figure 1A). After template design, circRNA^D2GFP^ was produced through splicing reaction. Samples were reverse transcribed and junction-spanning primers were utilized to amplify the product. Sanger sequencing was performed, which confirmed that circRNA^D2GFP^ was formed (Figure 1A). RNase R digestion assay also indicated the formation of circRNA^D2GFP^ after splicing reaction (Figure S1A). The final products were purified via liquid chromatography **(**Figure S1B**)** as referring to previous studies 31, 32. Human HEK293T cells and murine NIH3T3 cells were used for *in vitro* assay. We observed that D2GFP was more long-lasting in the circRNA^D2GFP^ group compared with the unmodified mRNA^D2GFP^ and M1Ψ mRNA^D2GFP^ groups (Figure 1B-C and Figure S1C-D). Statistical results also indicated that the ratio of D2GFP positive cells in circRNA^D2GFP^ groups was significantly higher than the other groups both at 48 hours and 72 hours after transfection (Figure 1D-E).

### Preparation of circRNA-LNP complex

**Figure 2:**
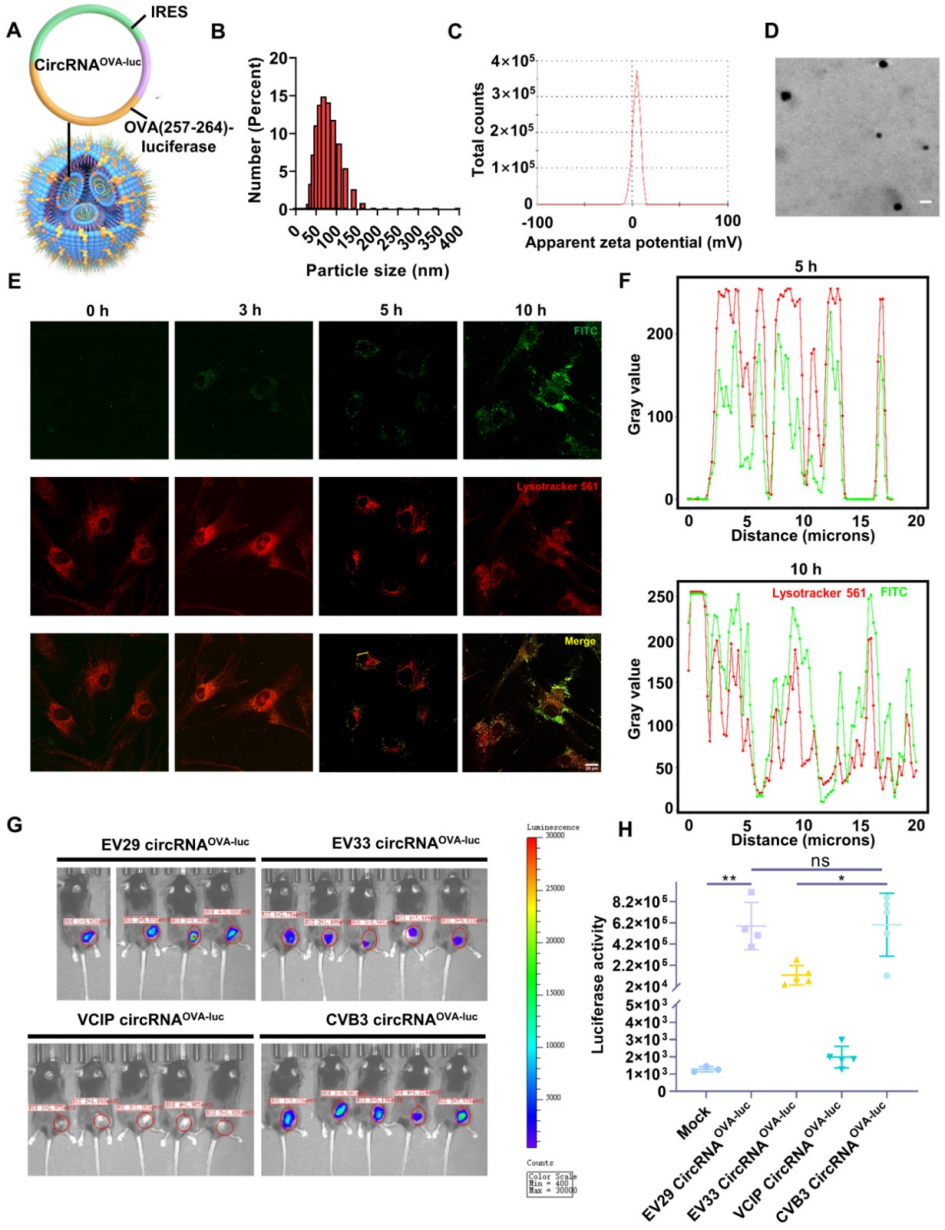
***In vitro* and *in vivo* characterization of the circRNA-LNP complex. (A)** Schematic representation of the circRNA^OVA-luc^-LNP complex. CircRNA encoding the restricting H2-Kb peptide OVA 257-264 and luciferase was encapsuled with LNP. **(B)** Size distributions of the circRNA^OVA-luc^-LNP complex. **(C)** Zeta potential of the circRNA^OVA-luc^-LNP complex. **(D)** TEM image of the circRNA^OVA-luc^-LNP complex. Scale bar, 100 nm. **(E)** Intracellular localization of the FITC-labeled LNP characterized by CLSM. Scale bar, 20 µm. **(F)** Analysis of the fluorescent value along the selected line (the yellow line) in the merged images. **(G)** Bioluminescence images of the mice after circRNA^OVA-luc^-LNP vaccination. CircRNA^OVA-luc^-LNP with different IRES elements were given intramuscularly to C57/B6 mice (10 µg circRNA per mouse), and luciferase expression was measured 6 hours post injection. **(H)** Statistical analysis for *in vivo* luciferase expression. ns, no significant, *p < 0.05, **p < 0.01.

Then, we developed a LNP system to investigate the function of circRNA vaccine *in vivo* (Figure S2A-B). The OVA (257-264)-luciferase-coding circRNA (circRNA^OVA-luc^) was purified (Figure S2C) and encapsulated by LNP to form a stable complex (Figure 2A), which was suitable for *in vivo* transfection. The average diameter of circRNA^OVA-luc^ LNP was measured to be 74 nm by dynamic light scattering (Figure 2B), and the corresponding zeta potential was approximately 4.09 mV (Figure 2C). The morphology of circRNA^OVA-luc^ LNP was also characterized via transmission electron microscopy, spherical nanoparticles were further confirmed (Figure 2D). Confocal microscopy (CLSM) was utilized to examine the intracellular trafficking profile of the complex by labeling the LNP with a fluorescence dye (Figure 2E-F). The image indicated that part of the complex entered the cells after three hours incubation. Five hours later, most of complex were visualized at lysosome, and the complex started to escape from the lysosome after ten hours incubation. Similar results were obtained in mouse embryonic fibroblasts (Figure S3A-C). Then, we encapsuled circRNA^D2GFP^ in LNP for transfection *in vitro* (Figure S4A). The result showed that circRNA was successfully delivered into cells and translated into protein. The biocompatibility of this LNP delivery system was verified by a CCK-8 assay, no significant change in the cell viability and proliferation was monitored even at high-dose treatment (Figure S4B).

As IRES-mediated translation is dependent on the availability of IRES trans-acting factors, which vary among cell and tissue types, different IRES elements or even the same IRES element initiate distinct translation efficiency 31, 34, 35. Thus, we aimed to select an IRES to optimize the protein expression of circRNA in mouse muscle tissues. Previous studies have provided some clues, CVB3-IRES, EV29-IRES, EV33-IRES, and VICP-IRES directed translation more efficiently than the commonly used EMCV-IRES in specific tissues and cells 31, 34, 35. These four IRES elements mentioned above were tested *in vitro* and *in vivo*. CircRNA^OVA-luc^ with different IRES elements were packaged with LNP, and their translation efficiency was detected and compared. For *in vitro* screening, the expression of firefly luciferase in EV29, EV33, and CVB3 groups was significantly higher than those in VCIP groups (Figure S4C-D). Moreover, *in vivo* comparison was performed using IVIS bioluminescent imaging (Figure 2G), the statistical analysis suggested that EV29 and CVB3 IRES structures offered better translation initiating efficiency than those of EV33 and VCIP elements (Figure 2H). These results indicated that both EV29 and CVB3 were ideal elements for circRNA^OVA-luc^ vaccination in mouse muscle tissue, and we chose CVB3 IRES for the following studies.

### CircRNA-LNP vaccine induces potent immunity indispensable for cancer immunotherapy

**Figure 3:**
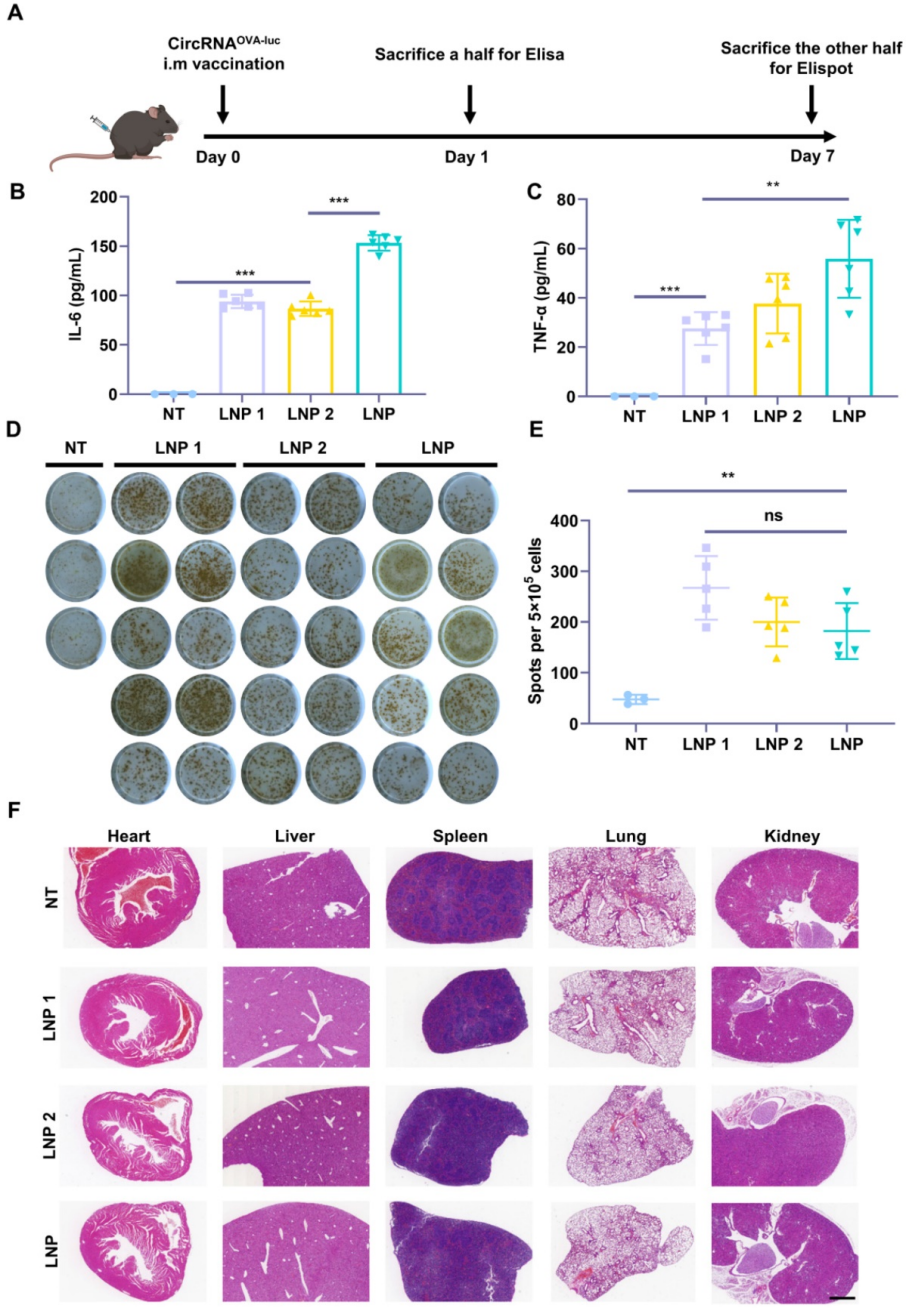
** Induction of innate and adaptive immune response by circRNA-LNP vaccine. (A)** Timeline of the experiment designed to evaluate the immune response triggered by the circRNA^OVA-luc^-LNP vaccine (10 µg circRNA per mouse). **(B-C)** Serum cytokine release after circRNA^OVA-luc^-LNP administration. Elisa assay was carried out to detect serum IL-6 and TNF-α secretion. **(D)** IFN-γ spot-forming cells and **(E)** statistical result from restimulated splenocytes detected via Elispot assay. **(F)** H&E staining for vital organs of the non-treated (NT) and three vaccinated groups with circRNA^OVA-luc^-LNP. Scale bar, 1 mm. *p < 0.05, **p < 0.01, ***p < 0.001.

As both innate and adaptive immune responses are essential for antitumor immunity, we next investigated the stimulatory capacity of innate and adaptive immune responses triggered by the circRNA^OVA-luc^-LNP complex (Figure 3A). Two FDA-approved LNP delivery systems (LNP 1 36 and LNP 2 37) were used as control groups (Figure S5A-B). All of the three circRNA^OVA-luc^-LNP vaccines showed similar particle size, zeta potential, and transfection efficiency *in vitro* (Figure 2B-C and Figure S5C-H). Mice were intramuscularly administrated with the circRNA^OVA-luc^-LNP vaccines, and peripheral blood was extracted for Elisa assay to detect the pro-inflammatory cytokines at 24 h post injection. As presented in Figure 3B**-**C, the innate immune responses were observed as illustrated by serum IL-6 and TNF-α secretion in all the circRNA^OVA-luc^-LNP groups. Notably, our LNP delivery system using a multi-armed ionizable lipid induced a significant increase in cytokine levels compared with other groups. To detect antigen-specific T cell response, mice were sacrificed and spleens were taken for Elispot assay. Figure 3D-E indicated that all the three circRNA^OVA-luc^-LNP vaccines induced robust antigen-specific T cell responses. The systemic toxicity of these vaccines was assessed by hematoxylin and eosin (H&E) staining of the main organs, no apparent toxic side effect was observed (Figure 3F). Furthermore, the innate immune response induced by circRNA^OVA-luc^-LNP complex and m1Ψ mRNA^OVA-luc^-LNP complex were also tested, and no significant differences were found between the two groups (Figure S6A-B).

### CircRNA-LNP vaccine mediates effective anti-tumor response in an immune-excluded subcutaneous tumor model

**Figure 4:**
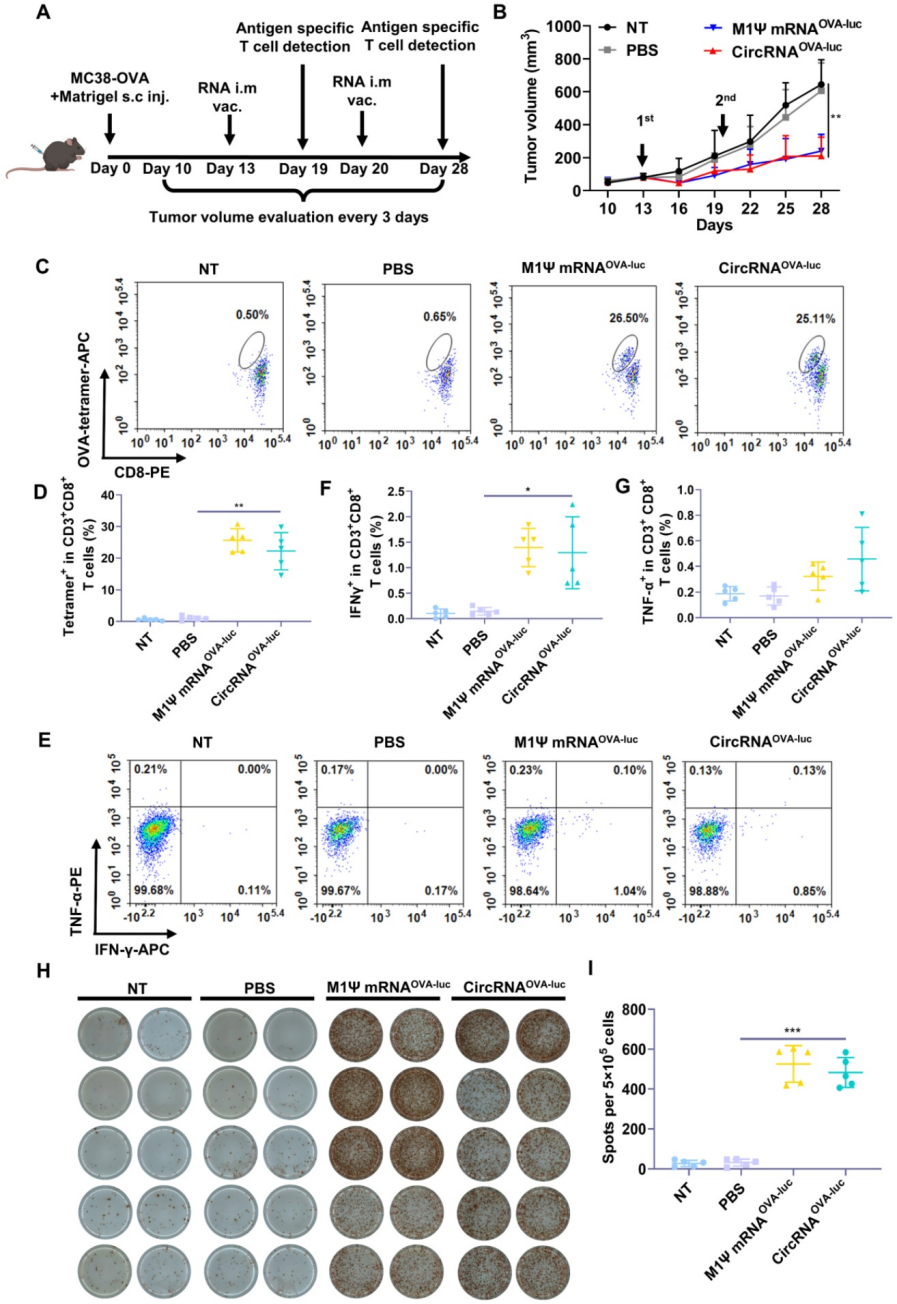
** CircRNA-LNP vaccine inhibits the tumor growth and drives potent antigen specific T cell response in MC38 subcutaneous tumor model. (A)** Timeline of the experiment to evaluate the anti-tumor efficacy of circRNA^OVA-luc^-LNP vaccine in MC38 subcutaneous injection model, intramuscular vaccination (10 µg circRNA per mouse). **(B)** Average tumor growth curve of the mice after different administrations (*n* = 5). NT, not treated; PBS, PBS-LNP complex; M1Ψ mRNA^OVA-luc^, M1Ψ mRNA^OVA-luc^-LNP complex; circRNA^OVA-luc^, circRNA^OVA-luc^-LNP. **(C)** Representative flow dot plots and **(D)** statistical result of the H-2Kb/SIINFEKL tetramer-positive T cells in PBMCs on day 6 post first immunization. **(E)** Representative flow dot plots and statistical data of the percentage of **(F)** IFN-γ and **(G)** TNF-α positive CD8^+^ T cells in the spleen at day 28. **(H)** IFN-γ spot-forming cells and **(I)** statistical data from restimulated splenocytes determined via Elispot assay at day 28. *p < 0.05, **p < 0.01, ***p < 0.001.

Encouraged by the impressive results, we evaluated the anti-tumor efficacy of RNA-LNP vaccines by establishing an immune-excluded murine tumor model using MC38-OVA cells. To mimic the dense extracellular matrix, a reconstituted extracellular matrix was mixed with tumor cells and subcutaneously administrated into the mice. The scheme of the experiment was performed in Figure 4A. After two doses of vaccination, both the circRNA-LNP^OVA-luc^ and m1Ψ mRNA^OVA-luc^-LNP significantly suppressed the tumor growth Figure 4B. During the treatment period, vaccination caused moderate weight loss in all the groups but all the mice regained much of the weight within six days (Figure S7A). Six days post first administration, the adaptive immune response was measured via flow cytometry. Notably, the frequency of SIINFEKL-MHC-I tetramer-positive cytotoxic T cells was significantly increased compared with the control groups, demonstrating the robust antigen-specific T cell response triggered by RNA-LNP vaccines (Figure 4C-D and Figure S7B). Generally, the second injection of vaccines helps to maintain the immune response for a longer period. As a result, mice were sacrificed and spleens were collected for analysis eight days after the second vaccination. Splenocytes were exposed to the same antigen and the expression of IFN-γ and TNF-α was evaluated via flow cytometry (Figure 4E-G and Figure S8). Elispot assay was also performed to verify the IFN-γ expression (Figure 4H-I). The secretion of IFN-γ was significantly increased in both RNA-LNP groups. For TNF-α detection, circRNA^OVA-luc^-LNP vaccine slightly enhanced the expression compared with m1Ψ mRNA^OVA-luc^-LNP group.

### CircRNA-LNP vaccine suppresses tumor progression in immune-desert orthotopic and metastasis melanoma model

**Figure 5:**
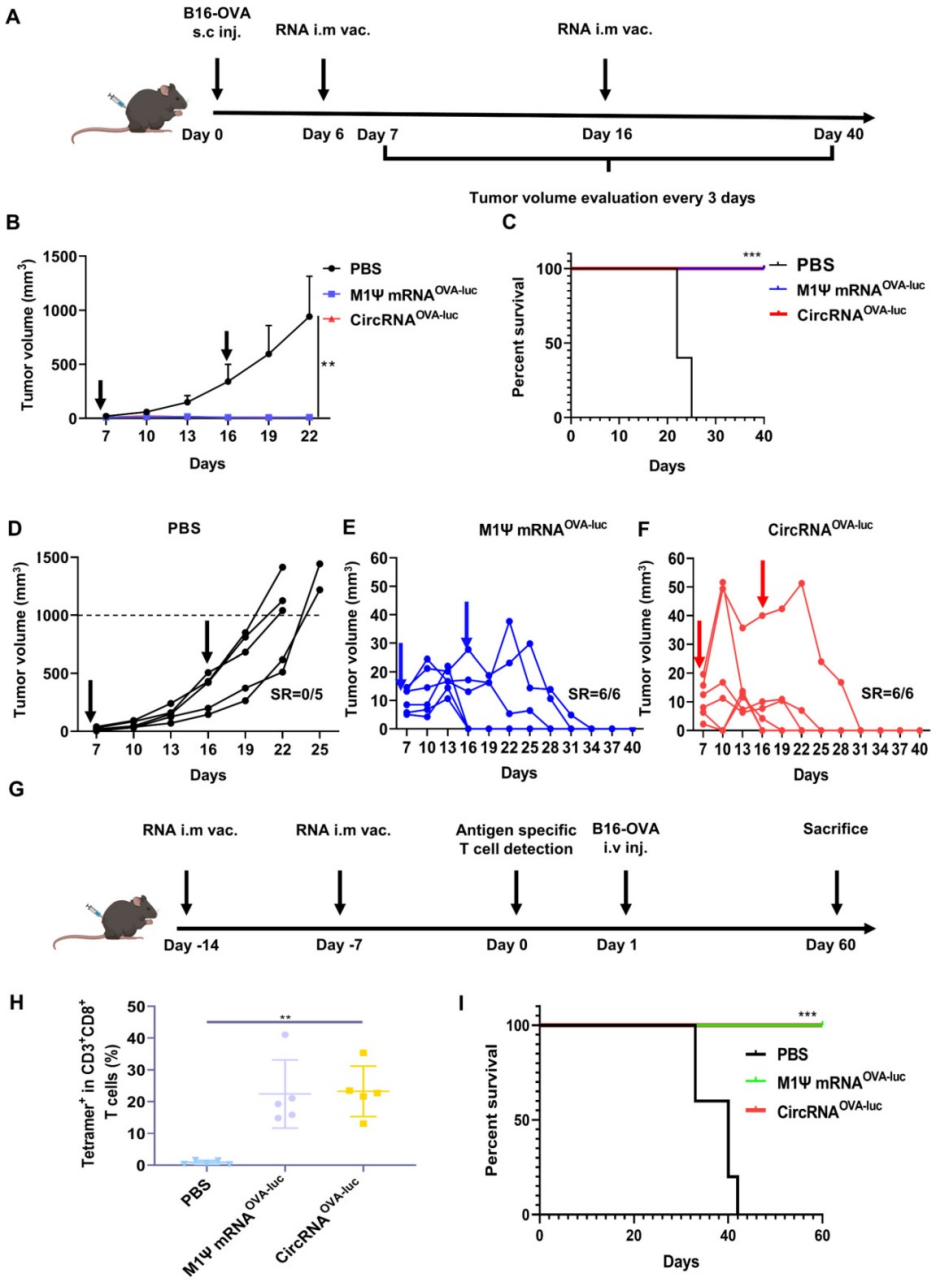
** CircRNA-LNP vaccine suppresses the progression in orthotopic and metastasis B16 tumor model. (A)** Timeline of the anti-tumor assay in orthotopic B16 tumor model, intramuscular vaccination (10 µg circRNA per mouse). **(B)** Tumor growth curves and **(C)** the survival curves of the three groups. PBS, PBS-LNP complex (*n* = 5); M1Ψ mRNA^OVA-luc^, M1Ψ mRNA^OVA-luc^-LNP complex (*n* = 6); circRNA^OVA-luc^, circRNA^OVA-luc^-LNP complex (*n* = 6). **(D-F)** Individual tumor growth curves of the mice from different groups. **(G)** Timeline of the anti-tumor assay in metastasis B16 tumor model, intramuscular vaccination (10 µg circRNA per mouse). **(H)** Statistical result of the H-2Kb/SIINFEKL tetramer-positive T cells in PBMCs seven days after the second immunization. PBS, PBS and LNP complex (*n* = 5); M1Ψ mRNA^OVA-luc^, M1Ψ mRNA^OVA-luc^-LNP (*n* = 5); circRNA^OVA-luc^, circRNA^OVA-luc^-LNP (*n* = 5). **(I)** Survival curve of mice after different treatments. **p < 0.01, ***p < 0.001.

A non-immunogenic metastasis and orthotopic tumor model were further established to evaluate the outstanding anti-tumor performance of RNA-LNP vaccines. For the therapeutic model of orthotopic inoculation, mice were subcutaneously injected with B16-OVA cells and received RNA-LNP vaccines on day 6 and 15 (Figure 5A). Surprisingly, vaccination of both circRNA^OVA-luc^-LNP and M1Ψ mRNA^OVA-luc^-LNP completely suppressed the tumor growth (Figure 5B). During tumor volume measurement, mice were defined as death when the volume reached 1000 mm^3^. Mice survival was analyzed based on the above principle (Figure 5C). All the mice died within 25 days after tumor inoculation, while all the mice survived in the groups vaccinated with RNA-LNP. Figure 5D-F showed the tumor growth curves of each group. Tumor volume raised dramatically in the control group, whereas the tumors reduced to an undetectable level in the vaccinated groups. Body weight was also monitored during the treatments (Figure S9A), and the result was in line with our previous observations. To investigate whether the RNA-LNP vaccines could block the formation of lung metastasis, mice were first immunized with two doses of RNA-LNP vaccines and challenged with B16-OVA cells via tail vein injection (Figure 5G). One day before tumor cell injection, antigen-specific T cells were determined in the peripheral blood. The percentage of SIINFEKL-MHC-I tetramer-positive cytotoxic T cells was significantly increased (Figure 5H and Figure S9B-C), which indicated that vaccination has already provided immune protection. Consistently, all the vaccinated mice survived two-month post tumor cell injection (Figure 5I). These results demonstrated that the immune response, and anti-tumor efficacy triggered by circRNA^OVA-luc^-LNP and M1Ψ mRNA^OVA-luc^-LNP showed no significant difference, indicating that the circRNA vaccine was an ideal alternative to the modified mRNA vaccine for cancer treatment.

### CircRNA-LNP vaccine induces completely tumor regression in late-stage tumor model via synergizing with adoptive cell transfer therapy

**Figure 6:**
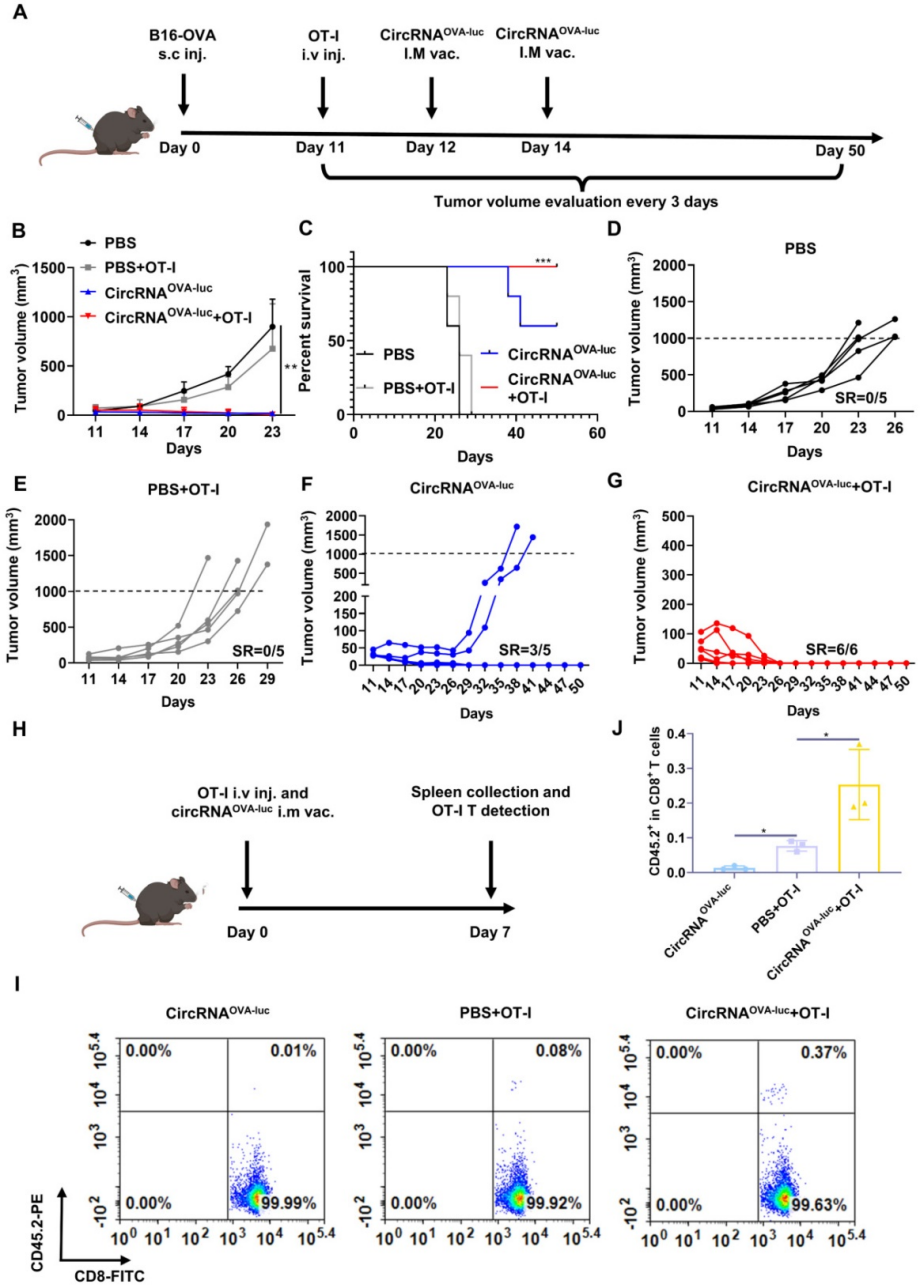
** CircRNA-LNP vaccine exerts synergistic efficacy with OT-I T cell transfer in late-stage B16 model. (A)** Timeline of the experiment to evaluate the synergetic anti-tumor effect of vaccine (circRNA^OVA-luc^) and TCR-T adoptively therapy (OT-I) in late-stage orthotopic B16 tumor model, intramuscular vaccination (5 µg circRNA per mouse), intravenous administration (5×10^5^ OT-I T cells per mouse). Average tumor growth curve** (B)** and the survival curve **(C)** of the four groups. PBS, PBS-LNP complex (*n* = 5); PBS+OT-I, PBS-LNP complex with OT-I transfer (*n* = 5), circRNA^OVA-luc^, circRNA^OVA-luc^-LNP complex (*n* = 5); circRNA^OVA-luc^+OT-1, circRNA^OVA-luc^-LNP with OT-I transfer (*n* = 6). **(D-G)** Individual tumor growth curves for four mouse groups. SR, survival rate. **(H)** Timeline of the experiment of OT-I T cell persistence evaluation. CD45.1 C57BL/6J mice were administrated with CD45.2 OT-I cells and circRNA^OVA-luc^-LNP (circRNA^OVA-luc^), intramuscular vaccination (5 µg circRNA per mouse), intravenous administration (1×10^6^ OT-I T cells per mouse). Seven days later, spleens were collected for analysis. Representative flow dot plots **(I)** and statistical result **(J)** of the OT-I (CD45.2^+^) T cells in CD8^+^ cells. *p < 0.05, ***p < 0.001.

As patients typically exhibit high tumor burden when diagnosed, combination treatments are more ideal for eliminating late-stage tumors. ACT therapy is another promising option of immunotherapy but showed inadequate response against the advanced solid tumor 38. One of the major barriers was the absence of proliferation signals when the engineered T cells encountered the tumor cells in an immunosuppressive tumor microenvironment 39. We supposed cancer vaccine was able to stimulate the engineered T cells *in vivo*, which induced a synergistic anti-tumor effect. Following this lead, we generated a late-stage immune desert orthotopic model via B16-OVA cells (Figure 6A). Before treatment, all the tumors were allowed to grow for 11 days to simulate a late-stage melanoma malignancy. Then, to manifest the synergistic of RNA vaccine and adoptive cell transfer, four mice groups were respectively injected with a PBS placebo, transferred with OT-I cell, administrated with the circRNA^OVA-luc^-LNP vaccine, and transferred with OT-I together with a circRNA^OVA-luc^-LNP vaccine administration, which acted as a negative control, adoptive T cell-treated group, an RNA vaccine-treated group, and a combination therapy group. Figure 6B showed both circRNA vaccination and combination therapy triggered robust anti-tumor responses, while TCR-T therapy alone only provided a slight anti-tumor efficacy compared with the control group. Mice survival was also analyzed (Figure 6C), all the mice died within 29 days in the PBS group and OT-I group. Noteworthy, three of five mice in vaccinated group, and all the mice in the combination group survived over 60 days. The tumor growth curve in each group was also presented in Figure 6D-G. The tumors in the groups receiving PBS or TCR-T showed a rapid growth over the therapy period. Three of five mice in the vaccinated groups showed complete regression of tumors. Importantly, all the tumors disappeared completely in the combination group, suggesting that the combination of circRNA vaccine and TCR-T therapy was superior to monotherapy. In order to reveal the mechanisms underlying the synergistic effects of combined therapy, we transferred OT-I T cells to CD45.1^+^ C57BL/6J and vaccinated them with circRNA^OVA-luc^-LNP (Figure 6H). We found that the percentage of OT-I T cells was significantly higher in the combination group than that in the OT-I group (Figure 6I-J and Figure S10), thus demonstrating that circRNA-LNP indeed improved the persistence of TCR-T cells.

## Discussion

Different from previous studies which used chemical reaction or T4 ligase to generate circRNA molecule with stringent size restriction 40, we adopted a novel circRNA synthesis method that used a permuted intron-exon (PIE) element to generate circRNA via *in vitro* back-splicing 31. This method permits us to produce circRNA of enough length to incorporate highly efficient IRES elements and antigen coding sequences. The generation of targeting circRNA molecules is confirmed by the existence of translatable RNA molecules that can resist RNase R digestion demonstrating the stability of circRNA molecules. A few studies have shown that secreted proteins coding by circRNA resulted in more persistent levels compared with those coding by mRNA with N1-methylpseudouridine (M1Ψ) 31. However, few reports directly proved the persistence of intracellular circRNA itself. Here, we proved that the half-life of the circRNA molecule is much longer than that of the corresponding nucleoside modified linear RNA by employing a short-lived D2GFP protein, a perfect molecule to compare the persistence of protein-coding RNA molecules immediately. The better persistence and lower production cost of circRNA molecules make it a tempting alternative for nowadays base-modified linear RNA.

RNA vaccine demonstrated its ability to provide humoral immune protection against viral infection, and the circRNA vaccine has been reported to induce an immune response against the most contagious viral mutant strain 33. However, unlike prophylactic vaccines that are destinated to provoke antibody-based B cell response, cancer RNA vaccines are supposed to arouse tumor-killing response mainly exerted by cytotoxic T cells 14. According to previous research, a systematic immune response and a proinflammatory immune context are required to induce robust cytotoxic T cell response, and prolonged antigen presentation is preferred to sustain active tumor-killing T cell function 41. CircRNA can elongate the production of tumor antigen, thus prolonging the antigen presentation of antigen-presenting cells. However, the purified circRNA is reported to be less immunogenic 32 and cannot provide a proinflammatory microenvironment suitable for cytotoxic T cell activation. To solve this dilemma, we cooperated with a novel ionizable lipid that is capable of inducing proinflammatory cytokine release into our LNP carrier. This combination exerts the prolonged protein translation ability of circRNA and at the same time provides a proinflammatory immune context suitable for cytotoxic T cell activation.

For better therapeutic intervention design, tumors are characterized into generally three types according to their immune micro-environment, the “hot tumors” which are highly immunogenic, the “immune exclusive tumors” which prevent T cell infiltration, and the “immune desert tumors” which show low T cell response 39. Hot tumors show a high response rate to immunotherapies such as ICB, whereas cold tumors and immune-exclusive tumors exhibit poor responses to present immunotherapies. Besides, the metastasis of tumor cells is the main reason for tumor therapy failure and the ability to prevent tumor metastasis is a critical index for evaluating the efficacy of tumor therapy 42. To evaluate the efficacy of our circRNA-LNP complex comprehensively, we use MC38 cell line together with high concentration Matrigel to simulate immune exclusive tumor model and use B16 cell line to build an immune desert tumor model and imitate tumor cell metastasis. Our circRNA-LNP vaccine can greatly suppress the progression of immune-exclusive tumors, induce complete tumor regression in immune desert tumors, and prevent cancer cell metastasis, which demonstrates the efficacy of the cancer RNA vaccine over currently used cancer immune therapies.

Late-stage malignancies show poor response to mono cancer immunotherapy type, mainly contributed to the establishment of multiple mechanisms to suppress immune activation and escape from cytotoxic T cell attack. A combination of cancer therapies is intensively studied to treat late-stage malignancies 43. Previous research has successfully combined mRNA vaccine and CAR-T or ICB therapy to treat late-stage melanomas 14, 44-45. Here, our circRNA-LNP can also synergize with adoptive cell transfer therapy and completely suppress the progression of late-stage immune exclusive tumors by enhancing the persistence of TCR-T cells. The data were in consistent with a previous research that RNA vaccine could drive expansion and efficacy of CAR-T cells against solid tumors 45. They found that RNA vaccine induced the activation of CAR-T cells via mediated display of the CAR target on dendritic cells and promoted efficient *in vivo* expansion, superior functionality, and memory formation of CAR-T cells. The mechanisms partially explain the phenomena of enhanced persistence of TCR-T and superior anti-tumor effect in combination treatment groups. Taken together, these results demonstrated the versatility of RNA vaccines to serve as both a major therapeutic intervention and synergize with currently used cancer immune therapies to suppress the progression of late-stage malignancies.

In conclusion, we designed a circRNA-LNP platform for application in therapeutic cancer RNA vaccine. CircRNA exhibited higher stability and initiated a more durable protein expression than its linear counterpart *in vitro*. CircRNA-LNP triggered remarkable innate immune response and potent antigen-specific T cell response, which was comparable to the function of modified mRNA-LNP *in vivo*. CircRNA-LNP platform exhibited superb therapeutic efficacy in the treatments of “immune-excluded” MC38 tumor model and “immune-desert” B16 orthotopic melanoma. The efficacy of circRNA-LNP vaccine was further verified in a B16 lung metastasis model. Moreover, this platform showed synergic anti-tumor effect with adoptive T cell therapy for late-stage B16 orthotopic melanoma model. These results demonstrated that circRNA-LNP vaccine could provoke extraordinary anti-tumor performances in various mouse tumor models. Due to its high stability, simple and economic manufacturing procedure, the circRNA-LNP platform holds the promise to become an attractive alternative for vaccination, which exhibits great potentials in clinical translation.

## Materials and methods

### Preparation of *in vitro* transcription (IVT) templates

For circRNA backbone, the T7 promoter, homology arm, elements from permuted intron-exon (PIE) construct, spacer, IRES, and coding sequences were synthesized by General Biol and cloned into puc57 plasmid. For linear RNA backbone, the T7 promoter, 5' untranslated region (UTR), Kozak sequence, coding sequences, and 3' UTR were synthesized by General Biol and cloned into puc57 plasmid. All the sequences coding D2GFP or OVA (257-264; SIINFEKL)-luciferase are supplied in Supplemental Table 1. Then, the plasmids were linearized by PCR amplification. PCR products were purified by agarose electrophoresis and gel extraction and indicated fragments were concentrated to more than 100 ng/µL in RNase-free water. After sequencing verification, the products were stored at -20 °C and were used as templates for IVT.

### Production and purification of RNA

For circRNA and unmodified mRNA, an IVT assay was carried out by using the T7 High Yield RNA Transcription Kit (Vazyme) following the manufacturer's protocol. The RNA was purified via DNase I digestion for 15 min at 37 °C, followed by lithium chloride precipitation. To obtain circRNA via splicing reaction, RNA was heated at 65 °C for 3 min and then immediately placed on ice, after that GTP (Invitrogen) was added to a final concentration of 2 mM and the reaction was carried out in T4 RNA Ligase Reaction Buffer (NEB) at 55 °C for 15 min. Products were then purified via liquid chromatography using a 7.8×300 mm size-exclusion column with a particle size of 5 μm and pore size of 2000 Å (Sepax Technologies, 215980-7830). Briefly, RNA was heated at 65 °C for 3 min and then immediately placed on ice. Next, RNA was run on an AKTA purifier system in RNase-free TE buffer (pH = 6.0). The enriched circRNA was subsequently purified using ammonium acetate precipitation and resuspended in RNase-free water. For N1-methylpseudouridine (m1Ψ) modified mRNA, an IVT assay was carried out by using the T7 High Yield RNA Transcription Kit (N¹-Me-Pseudo UTP) following the manufacturer's protocol and purified via lithium chloride precipitation. Then, mRNA was heated at 65 °C for 3 min and then immediately placed on ice, after which mRNA was capped using the Vaccinia Capping System (Vazyme) and the Cap 2'-O-Methyltransferase (Vazyme) according to the manufacturer's protocols. The products were purified via lithium chloride precipitation and polyA-tailing was performed via the E.coli Poly (A) Polymerase (Novoprotein). The fully processed RNA was purified via lithium chloride precipitation and resuspended in RNase-free water. For RNase digestion assay. To confirm the generation of circRNA via splicing reaction, RNA samples were digested by RNase R (Epicentre) at 37 °C for 15 min, followed by agarose gel electrophoresis. RNA samples were also reverse transcribed to cDNA and then amplified via PCR by junction-spanning primers. After that the sample was sent for Sanger sequencing.

### Mouse and cells

The female C57BL/6J mice (6 to 8 week old) were purchased from Beijing Vital River Laboratory Animal Technology. The OT-I mice were kindly provided by Chen Dong's lab (Tsinghua University). The female CD45.1 C57BL/6J mice were purchased from the laboratory animal resources center of Tsinghua University. All the mice were housed under SPF-grade conditions in the animal facility of Tsinghua University. All the animal experiments strictly adhered to the compliance standards of the Institutional Animal Care and Use Committee. HEK293T and NIH3T3 cells were purchased from American Type Culture Collection (ATCC). MC38-OVA cells were kindly provided by Rui Kuai's lab (Tsinghua University). B16-OVA cells were kindly provided by Meng Xu's lab (Tsinghua University). Cells were cultured in DMEM complete medium (with an addition of 10% fetal bovine serum (FBS), and 1% penicillin-streptomycin solution). Cell culture was undertaken in a CO_2_ incubator at 37 °C. For splenocytes culture, RPMI-1640 medium was supplemented with heat-inactivated 10% FBS and IL-2 (200 IU/ml).

### RNA transfection *in vitro*

Cells were seeded in 48-well plates. For each well, 200 nanograms of circRNA were transfected by using the Lipofectamine MessengerMax (Invitrogen) following the manufacturer's protocol. Equimolar quantities of mRNA were transfected via the same method. For the D2GFP assay, cells were collected at 24-, 48-, and 72-hours post-transfection and analyzed via flow cytometry. For luciferase activity detection, cells were collected 24 hours post-transfection, and luciferase activity was measured by a luciferase detection kit (Yeasen).

### Encapsulation of RNA by LNP

For the synthesis of multi-armed ionizable lipid, PAMAM dendrimer G0 was mixed with 1,2-epoxytetradecane at a molar ratio of 1:7. The mixture was reacted under vigorous stirring at 90 °C for 3 days. The crude reaction mixture was separated by chromatography on silica with gradient elution from CH_2_Cl_2_ to CH_2_Cl_2_/MeOH/NH_4_OH (75/22/3, *v*/*v*/*v*). LNP were prepared by combining an aqueous phase containing mRNA with an ethanol phase containing the lipid and cholesterol components via microfluidic mixing devices (Micro&Nano Technologies). The devices utilized chaotic mixing features to induce fluid folding in a state of laminar flow to reproducibly form homogeneous LNP. The aqueous phase was composed of 100 mM citrate buffer and mRNA. The ethanol phase contained the ionizable lipid, 1,2-distearoyl-sn-glycerol-3-phosphocholine (Sinopeg), cholesterol (Sinopeg), and lipid anchored polyethylene glycol (Sinopeg) at the molar ratio of 50:10:38:2. The aqueous and ethanol phases were then mixed in the microfluidic device at a 3:1 ratio.After synthesis, the LNP were dialyzed against PBS for 12 hours (MWCO = 3.5 kDa). For LNP-1 and LNP-2 used in this study, the ionizable lipid was replaced by two FDA-approved ionizable lipids (Supplementary Figure 5A-B, Sinopeg) and the manufacture steps are consistent with preparation methods of LNP. To measure size, the LNPs were suspended in PBS and analyzed using dynamic light scattering (DLS) performed on a Zetasizer Nano (Malvern Instruments, Malvern, UK). The diameter and polydispersity index of the LNP were measured in triplicate.

### Intracellular distribution of the LNP complex

For convenient imaging of the lysosome, mouse embryonic fibroblasts and human fibroblasts were cultured in glass-bottomed confocal dishes. Lysotracker 488 or 561 (Beyotime) were first incubated with cells for 20 minutes and changed with culture medium. Then R-300 or FITC-encapsuled LNP were added to cells and cells were imaged via confocal microscope (FV 3000RS, Nikon) at indicating time points. Co-localization was analyzed by Fiji software.

### *In vitro* cytotoxic assay

HEK293T cells and NIH3T3 cells were seeded in 96-well plate at a density of 5×10^4^/mL. Cells were cultured for 6 hours and circRNA-LNP complex were added to the cells at different doses. 24 hours after incubation, CCK-8 solution (Beyotime) was added to cells and incubated for another one hour and the absorbance was measured at 450 nm.

### *In vivo* RNA-LNP vaccine injection assay

For IRES screening assay, intramuscular (i.m) administrations of RNA-LNP (10 μg/mouse) or PBS-LNP complex were performed on the lateral side of the thigh. 6 hours post-injection, mice were injected intraperitoneally with D-luciferin (150 mg/kg, Yeasen). 5 minutes later, bioluminescence was measured by an IVIS Spectrum imaging system. For innate immune response evaluation, RNA-LNP or PBS-LNP was injected as described above. 24 hours post-injection, mice were sacrificed and peripheral blood was extracted and red blood cells were lysed with red blood cell lysis buffer. Subsequently, the serum was extracted for Elisa assay to detect the level of IL-6 and TNF-α (Thermo, 88-7064-88 and 88-7324-77) following the manufacturer's protocols. For HE-staining of mouse tissue, samples were collected, fixed and paraffin-embedded, sectioned, and stained for H&E (Servicebio).

### *In vivo* tumor models

For the metastasis model, CircRNA^OVA-luc^-LNP (10 μg circRNA per mouse), an equimolar amount of M1Ψ mRNA^OVA-luc^-LNP or LNP and PBS complex were intramuscularly injected into three parallel C57/B6 mouse groups at day -14 and day -7 to provide immune protection. Peripheral blood was extracted at day 0 for antigen-specific T cell detection. On day 1, 2×10^5^ B16-OVA cells were injected intravenously to simulate B16 tumor lung metastasis. The survival of mice was recorded for 60 days. To develop an immune exclusion tumor model, mice were subcutaneously injected with 5×10^5^ MC38-OVA cells together with Matrigel (Corning). The mix ratio of cells in PBS and Matrigel was 1:1 by volume. RNA-LNP (10 μg/mouse) or PBS-LNP were injected intramuscularly into mouse groups at day 13 and day 20 for therapeutic usage. Tumor volume was measured every 3 days since day 10 by Vernier Calipers. The tumor volume was calculated by the formation: *V* = length×width×width/2 (mm^3^). Peripheral blood was collected for antigen-specific T cell analysis via flow cytometry six days after the first vaccination. Eight days after the second vaccination, all the mice were sacrificed and the spleen was collected to evaluate the antigen-specific T cell via flow cytometry and Elispot. For the immune desert orthotopic tumor model, mice were subcutaneously injected with 5×10^5^ B16-OVA cells at day 0. circRNA-LNP (10 μg/mouse), an equal molar amount of M1Ψ mRNA-LNP or PBS-LNP were injected intramuscularly into mouse groups at day 6 and day 15 to stimulate anti-tumor immune responses. Tumor volume was measured and calculated as described above. Mice were sacrificed when the tumor volume reached 1,000 mm^3^. For the late-stage solid tumor model, C57BL/6 mice were subcutaneously injected with 5×10^5^ B16-OVA cells at day 0, the tumor was allowed to grow for 11 days which simulated a late-stage melanoma malignancy. Four groups of mice were injected with PBS-LNP intramuscularly, PBS-LNP intramuscularly, and OT-I T cells intravenously, circRNA^OVA-luc^-LNP intramuscularly, and circRNA^OVA-luc^-LNP intramuscularly and OT-I T cells intravenously to act as the control group, TCR-T adoptively therapy group, CircRNA vaccinated group and combined therapy group, respectively. Tumor volume was measured and calculated as described above. Mice were sacrificed when the tumor volume reached 1,000 mm^3^.

### Antigen-specific T cell analysis

For peripheral blood, samples were lysed with red blood cell lysis buffer and resuspended in a FACS buffer containing 1×PBS with 1% FBS and 1mM EDTA. Cells were blocked with an anti-mouse FC blocker (BD) at 4 °C for 20 min. After that cells were washed with FACS buffer once and stained with OVA Tetramer-SIINFEKL-APC (MBL, TS-5001-2C) at 4 °C for 30 min (protected from light). Next, cells were stained with Fixable Viability Dye eF506 (eBioscience, 65-0866-18), FITC anti-mouse CD3 (Biolegend, 100306), PE anti-mouse CD8 (Biolegend, 100708) at 4 °C for 30 min (protected from light). Finally, cells were washed with FACS buffer two times and fixed in 4% paraformaldehyde. Flow cytometry was carried out via a BD Fortessa or LSRII cytometer. Spleens were collected, ground, and filtered for splenocytes through a 75 µm screen. Red blood cells were lysed with red blood cell lysis buffer. Splenocytes were cultured at a 24-well plate (2×10^6^/well) for 6 hours with an addition of 10 μg/ml OVA 257-264 peptide (genscript, RP10611-10) and Golgi stop reagent (BD, 554715). Then, cells were stained with FITC anti-mouse CD3 (Biolegend, 100306), Pacific Blue anti-mouse CD8 (Biolegend, 100725), PE anti-mouse TNF-α(eBioscience, 12-7321-82), and APC anti-mouse IFN-γ (Biolegend, 505810) according to the protocol of Cytofix/Cytoperm Fixation/Permeabilization kit (BD, 554714). For the Elispot assay, splenocytes were cultured at a mouse IFN-γ precoated 96-well plate (5×10^5^ cells/well) with the addition of 10 μg/ml OVA 257-264 peptide (Genscript, RP10611-10) and cultured for 20 hours. After that, the assay was carried out using the Elispot kit (Dakewe Biotech, 2210005) following the manufacturer's instructions.

### OT-I cell transfer assay

CD45.2 OT-I mice were sacrificed and spleens were collected for T cell isolation following the protocol of EasySep™ Mouse T Cell Isolation Kit (Stemcell, 19851). Then, OT-I T cells (1×10^6^ per mouse, intravenous) with PBS-LNP, circRNA-LNP complex (5 μg circRNA per mouse, intramuscular) alone or OT-I T cells (1×10^6^ per mouse, intravenous) with circRNA-LNP complex (5 μg circRNA per mouse, intramuscular) were administrated to CD45.1 C57BL/6J mice. After seven days, mice were sacrificed and spleens were collected, ground and filtered through a 75 µm screen. Red blood cells were lysed with red blood cell lysis buffer. Then, cells were stained with FITC anti-mouse CD8 (eBioscience, 11-0081-82), Fixable Viability Dye eF780 (eBioscience, 65-0865-18) and PE anti-mouse CD45.2 (BD, 560695). Finally, cells were washed with FACS buffer two times and fixed in 4% paraformaldehyde. Flow cytometry was carried out via a BD Fortessa cytometer.

### Statistical analysis

All data in this study were analyzed via Graphpad Prism and presented as mean ±s.d. The unpaired t-test with Welch's correction was used for the two-group comparison. For survival curves, the data was performed via Kaplan-Meier analysis. The level of significance was defined at *p < 0.05, **p < 0.01, ***p < 0.001.

## Supplementary Material

Supplementary figures and table.

## Abbreviations

mRNA: messenger RNA
circRNA: circular RNA
LNP: lipid nanoparticle
ATC: adoptive cell transfer
IRES: internal ribosome entry site
PIE: permuted intron-exon

## References

[1] Zhang Y, Zhang Z (2020). The history and advances in cancer immunotherapy: understanding the characteristics of tumor-infiltrating immune cells and their therapeutic implications. Cell Mol Immunol.

[2] Chen XS, Moon JJ, Cheon J (2020). New Opportunities in Cancer Immunotherapy and Theranostics. Acc Chem Res.

[3] Wang J, Zhang X, Zhou Z, Liu Y, Yu L, Jia L A Novel Adoptive Synthetic TCR and Antigen Receptor (STAR) T-Cell Therapy for B-Cell Acute Lymphoblastic Leukemia. Am J Hematol, in press.

[4] Liu Y, Liu G, Wang J, Zheng ZY, Jia L, Rui W (2021). Chimeric STAR receptors using TCR machinery mediate robust responses against solid tumors. Sci Transl Med.

[5] Chen Z, Kankala RK, Yang Z, Li W, Xie S, Li H (2022). Antibody-based drug delivery systems for cancer therapy: Mechanisms, challenges, and prospects. Theranostics.

[6] Park JH, Rivière I, Gonen M, Wang X, Sénéchal B, Curran KJ (2018). Long-Term Follow-up of CD19 CAR Therapy in Acute Lymphoblastic Leukemia. N Engl J Med.

[7] June CH, O'Connor RS, Kawalekar OU, Ghassemi S, Milone MC (2018). CAR T cell immunotherapy for human cancer. Science.

[8] Dai H, Wang Y, Lu X, Han W (2016). Chimeric Antigen Receptors Modified T-Cells for Cancer Therapy. Natl Cancer Inst.

[9] Galon J, Bruni D (2019). Approaches to treat immune hot, altered and cold tumours with combination immunotherapies. Nat Rev Drug Discov.

[10] Chen DS, Mellman I (2017). Elements of cancer immunity and the cancer-immune set point. Nature.

[11] Newick K, O'Brien S, Moon E, Albelda SM (2017). CAR T Cell Therapy for Solid Tumors. Annu Rev Med.

[12] Beck JD, Reidenbach D, Salomon N, Sahin U, Türeci Ö, Vormehr M (2021). mRNA therapeutics in cancer immunotherapy. Mol Cancer.

[13] Pastor F, Berraondo P, Etxeberria I, Frederick J, Sahin U, Gilboa E (2018). An RNA toolbox for cancer immunotherapy. Nat Rev Drug Discov.

[14] Sahin U, Oehm P, Derhovanessian E, Jabulowsky RA, Vormehr M, Gold M (2020). An RNA vaccine drives immunity in checkpoint-inhibitor-treated melanoma. Nature.

[15] Liu C, Liu X, Xiang X, Pang X, Chen S, Zhang Y (2022). A nanovaccine for antigen self-presentation and immunosuppression reversal as a personalized cancer immunotherapy strategy. Nat Nanotechnol.

[16] Yang W, Zhu G, Wang S, Yu G, Yang Z, Lin L (2019). *In situ* Dendritic Cell Vaccine for Effective Cancer Immunotherapy. ACS Nano.

[17] Chu Y, Liu Q, Wei J, Liu B (2018). Personalized cancer neoantigen vaccines come of age. Theranostics.

[18] Shi Y, Liu Y, Huang J, Luo Z, Guo X, Jiang M (2022). Optimized mobilization of MHC class I- and II- restricted immunity by dendritic cell vaccine potentiates cancer therapy. Theranostics.

[19] Fritah H, Rovelli R, Chiang CL, Kandalaft LE (2022). The current clinical landscape of personalized cancer vaccines. Cancer Treat Rev.

[20] Miao L, Zhang Y, Huang L (2021). mRNA vaccine for cancer immunotherapy. Mol Cancer.

[21] He Q, Gao H, Tan D, Zhang H, Wang JZ (2022). mRNA cancer vaccines: Advances, trends and challenges. Acta pharmaceutica Sinica B.

[22] Li Y, Ma X, Yue Y, Zhang K, Cheng K, Feng Q (2022). Rapid Surface Display of mRNA Antigens by Bacteria-Derived Outer Membrane Vesicles for a Personalized Tumor Vaccine. Adv Mater.

[23] Pardi N, Hogan MJ, Porter FW, Weissman D (2018). mRNA vaccines - a new era in vaccinology. Nat Rev Drug Discov.

[24] Zeng C, Hou X, Yan J, Zhang C, Li W, Zhao W (2020). Leveraging mRNA Sequences and Nanoparticles to Deliver SARS-CoV-2 Antigens *In vivo*. Adv Mater.

[25] Jackson NAC, Kester KE, Casimiro D, Gurunathan S, DeRosa F (2020). The promise of mRNA vaccines: a biotech and industrial perspective. NPJ vaccines.

[26] Lasse SK, Maria SA, Lotte VWS, Karoline KE, Thomas BH, Jørgen K (2019). The biogenesis, biology and characterization of circular RNAs. Nature Reviews Genetics.

[27] Ling-Ling C (2016). The biogenesis and emerging roles of circular RNAs. Nat Rev Mol Cell Biol.

[28] Patop IL, Wüst S, Kadener S (2019). Past, present, and future of circRNAs. EMBO J.

[29] Kristensen LS, Andersen MS, Stagsted LVW, Ebbesen KK, Hansen TB, Kjems J (2019). The biogenesis, biology and characterization of circular RNAs. Nat Rev Genet.

[30] Lei M, Zheng G, Ning Q, Zheng J, Dong D (2020). Translation and functional roles of circular RNAs in human cancer. Mol Cancer.

[31] Wesselhoeft RA, Kowalski PS, Anderson DG (2018). Engineering circular RNA for potent and stable translation in eukaryotic cells. Nat Commun.

[32] Wesselhoeft RA, Kowalski PS, Parker-Hale FC, Huang Y, Bisaria N, Anderson DG (2019). RNA Circularization Diminishes Immunogenicity and Can Extend Translation Duration *In vivo*. Mol Cell.

[33] Qu L, Yi Z, Shen Y, Lin L, Chen F, Xu Y (2022). Circular RNA vaccines against SARS-CoV-2 and emerging variants. Cell.

[34] Yang J, Zhu J, Chen Y, Du Y, Tan Y, Wu L (2021). Intratumoral Delivered Novel Circular mRNA Encoding Cytokines for Immune Modulation and Cancer Therapy. bioRxiv. 2021.

[35] Licursi M, Christian SL, Pongnopparat T, Hirasawa K (2011). *In vitro* and *in vivo* comparison of viral and cellular internal ribosome entry sites for bicistronic vector expression. Gene Ther.

[36] Baden LR, El Sahly HM, Essink B, Kotloff K, Frey S, Novak R (2021). Efficacy and Safety of the mRNA-1273 SARS-CoV-2 Vaccine. N Engl J Med.

[37] Skowronski DM, De Serres G (2021). Safety and Efficacy of the BNT162b2 mRNA Covid-19 Vaccine. N Engl J Med.

[38] Majzner RG, Mackall CL (2019). Clinical lessons learned from the first leg of the CAR T cell journey. Nat Med.

[39] Anandappa AJ, Wu CJ, Ott PA (2020). Directing Traffic: How to Effectively Drive T Cells into Tumors. Cancer Discov.

[40] Petkovic S, Müller S (2015). RNA circularization strategies *in vivo* and *in vitro*. Nucleic Acids Res.

[41] Bhardwaj N (2007). Harnessing the immune system to treat cancer. J Clin Invest.

[42] Fares J, Fares MY, Khachfe HH, Salhab HA, Fares Y (2020). Molecular principles of metastasis: a hallmark of cancer revisited. Signal Transduct. Target Ther.

[43] Tan AC, Bagley SJ, Wen PY, Lim M, Platten M, Colman H (2021). Systematic review of combinations of targeted or immunotherapy in advanced solid tumors. J Immunother Cancer.

[44] Wang Y, Zhang L, Xu Z, Miao L, Huang L (2018). mRNA Vaccine with Antigen-Specific Checkpoint Blockade Induces an Enhanced Immune Response against Established Melanoma. Mol Ther.

[45] Reinhard K, Rengstl B, Oehm P, Michel K, Billmeier A, Hayduk N (2020). An RNA vaccine drives expansion and efficacy of claudin-CAR-T cells against solid tumors. Science.

