# Supplementary material for: Circular RNA cancer vaccines drive immunity in hard-to-treat malignancies
Source: Theranostics. 2022 Aug 29;12(14):6422–36. doi: 10.7150/thno.77350 (PMC9475446; doi:10.7150/thno.77350)
Supplement: Supplementary file 1 — Supplementary figures and table. [file thnov12p6422s1.pdf]

Circular RNA cancer vaccines drive immunity in hard-to-treat malignancies

Hongjian Li[1†], Kun Peng[1†], Kai Yang[2], Wenbo Ma[1], Shaolong Qi[2], Xinyang Yu[2], Jia He[3], Xin Lin[1,4]*, and Guocan Yu[2]*

[1]*Institute for Immunology and School of Medicine, Tsinghua University, Beijing 100084, China.*

[2]*Key Laboratory of Bioorganic Phosphorus Chemistry & Chemical Biology, Department of Chemistry, Tsinghua University, Beijing, 100084, China.*

[3]*School of Pharmaceutical Sciences, Tsinghua University, Beijing 100084, China.*

[4]*Tsinghua-Peking Center for Life Sciences, Beijing 100084, China.*

[†]These authors contributed equally to the manuscript.

* Correspondence:

Guocan Yu (guocanyu@mail.tsinghua.edu.cn);

Xin Lin (linxin307@tsinghua.edu.cn)

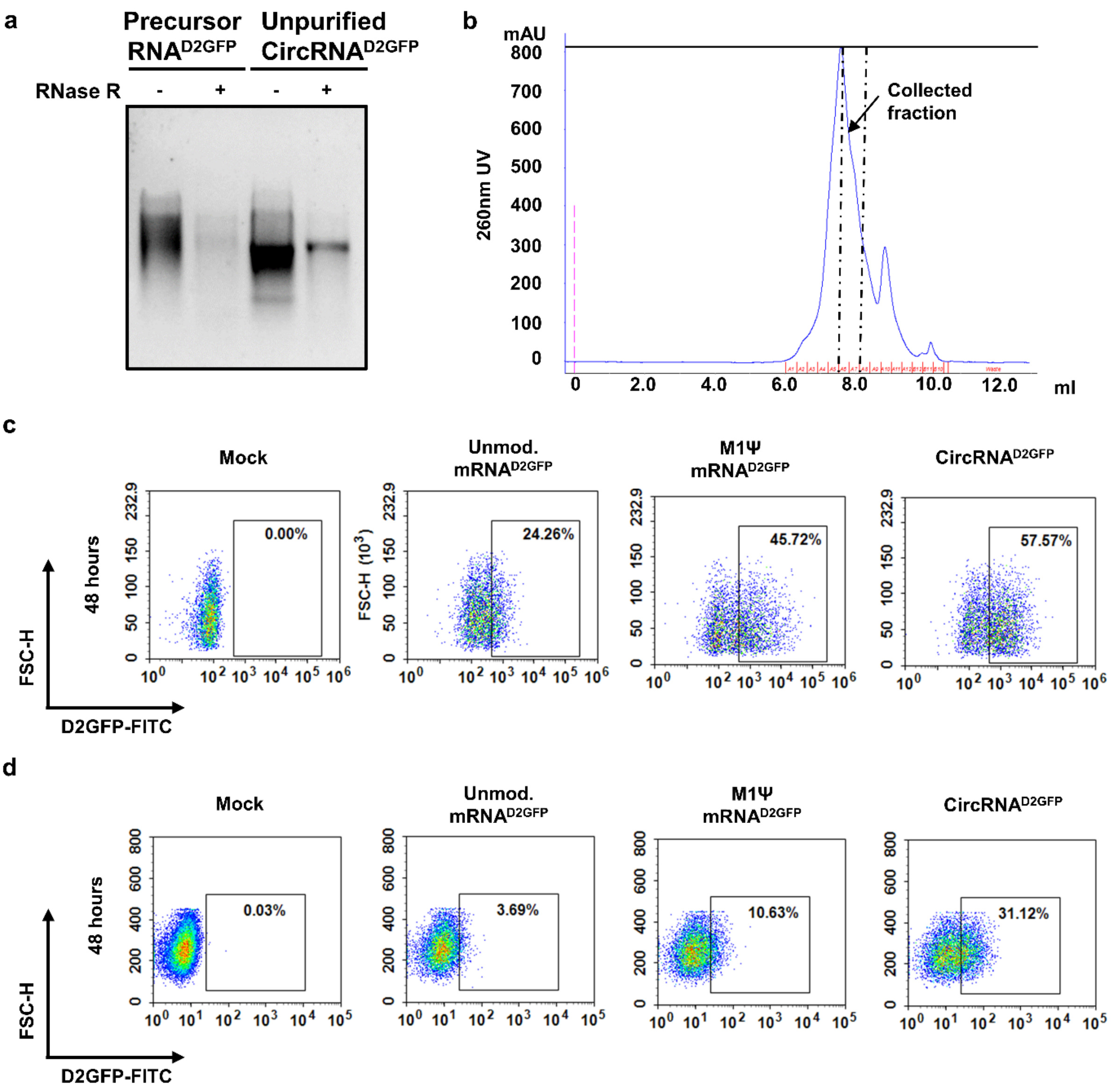

Figure 1 **(a)** Agarose gel electrophoresis of D2GFP-coding circRNA precursor (precursor RNA^{D2GFP}) and unpurified circRNA (unpurified $circRNA^{D2GFP}$) after RNase R treatment. **(b)** Chromatogram of D2GFP-coding $circRNA^{D2GFP}$ via an AKTA purifier system. Protein expression level of $circRNA^{D2GFP}$ and linear RNA^{D2GFP} in HEK293T cells **(c)** and NIH3T3 cells **(d)** 48 hours after transfection.

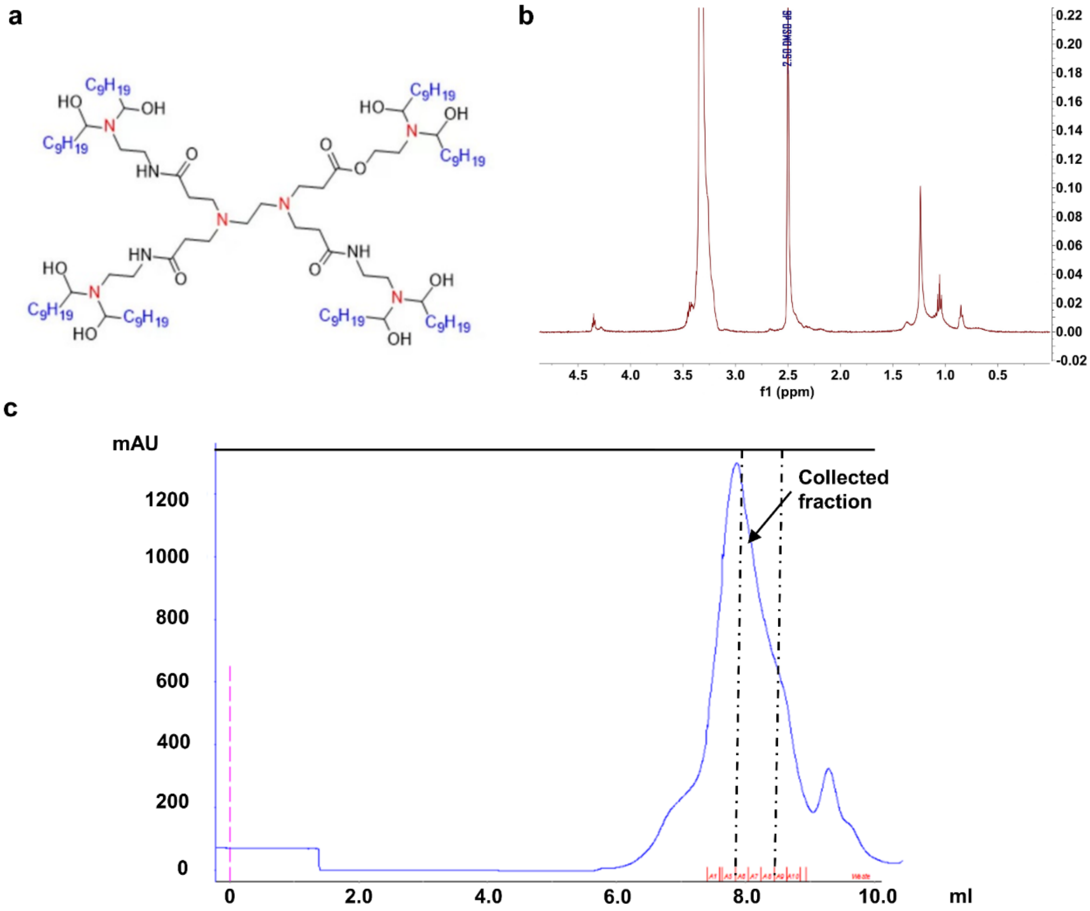

Figure 2 (a) The molecular structural formula of the novel ionizable lipid. **(b)** ^{1}H NMR spectrum of the compound. **(c)** Chromatogram of OVA (257-264)-luciferase-coding circRNA ($circRNA^{OVA\text{-}luc}$) via an AKTA purifier system.

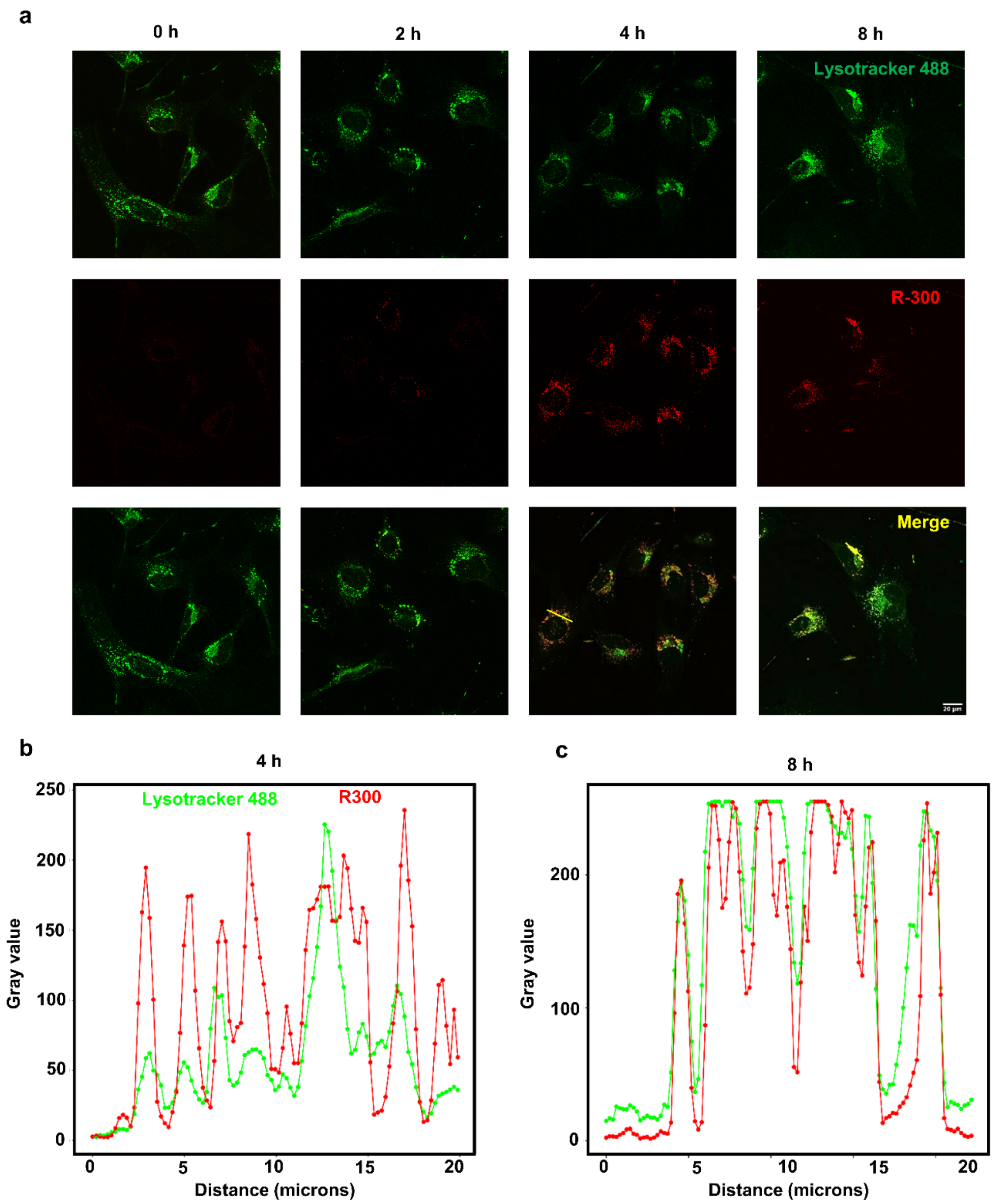

Figure 3 **(a)** Intracellular localization of the LNP and lysosome, characterized by confocal fluorescence microscopic imaging. Red fluorescence dye R300 was encapsulated in LNP and then incubated with mouse embryonic fibroblasts for 0, 2, 4 and 8 hours. Green, Lysotracker 488. Scale bar, 20μm. **(b)** Analysis of the fluorescent value along the selected line (the yellow line) in the merged images.

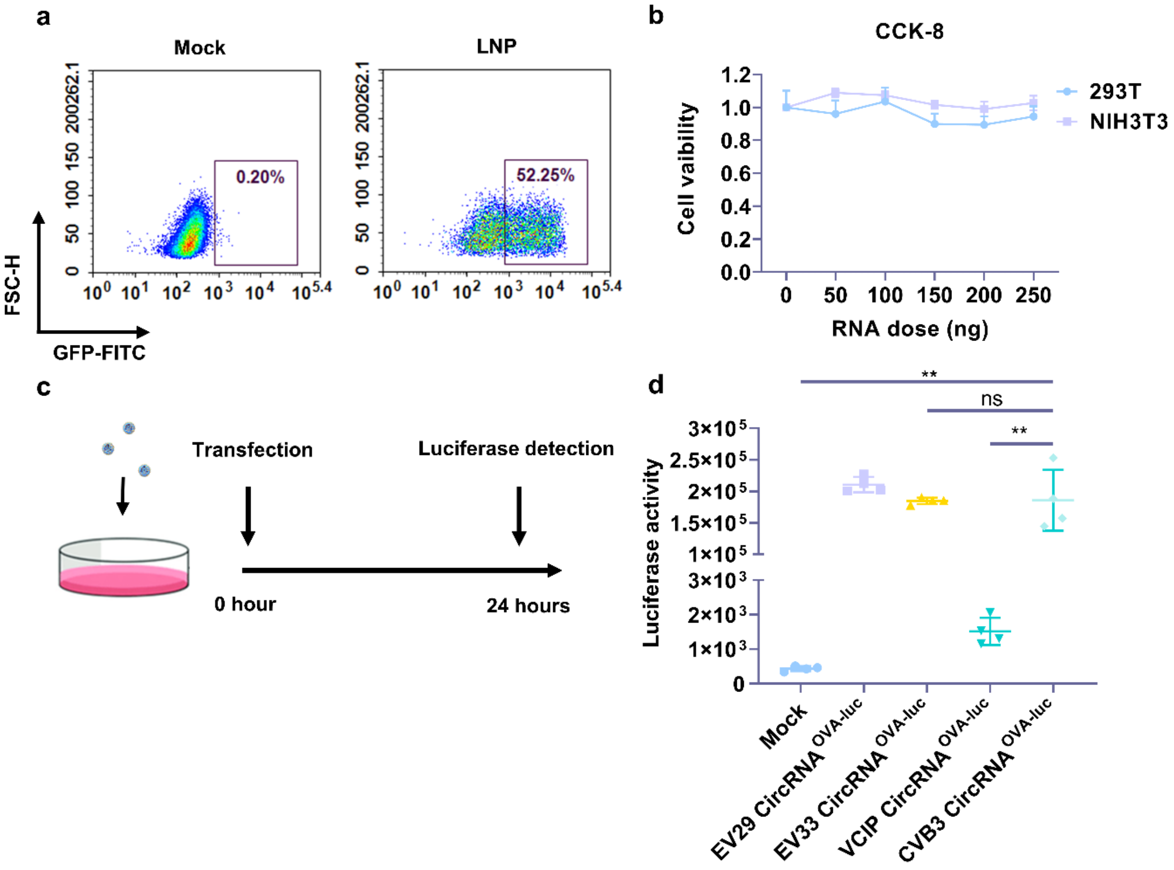

Figure 4 (a) Flow cytometric analysis of D2GFP expression in HEK293T cells. $CircRNA^{D2GFP}$ was encapsulated with LNP for transfection. 24 hours after transfection, cells were harvested for flow cytometry assay. **(b)** Cell viability after treatment with increasing doses of the $circRNA^{D2GFP}$-LNP complex. Cells were transfected with increasing RNA doses via LNP. After 24 hours, cell viability was evaluated via CCK-8 assay. **(c)** A workflow of the IRES screening assay in vitro. OVA (257-264)-luciferase-coding circRNA ($circRNA^{OVA\text{-}luc}$) with different IRES elements was packaged with LNP and transfected into HEK 293T cells, and luciferase activity was measured 24h after transfection. **(d)** Statistical analysis of luciferase activity in vitro. ns, no significant, $^{**}p < 0.01$.

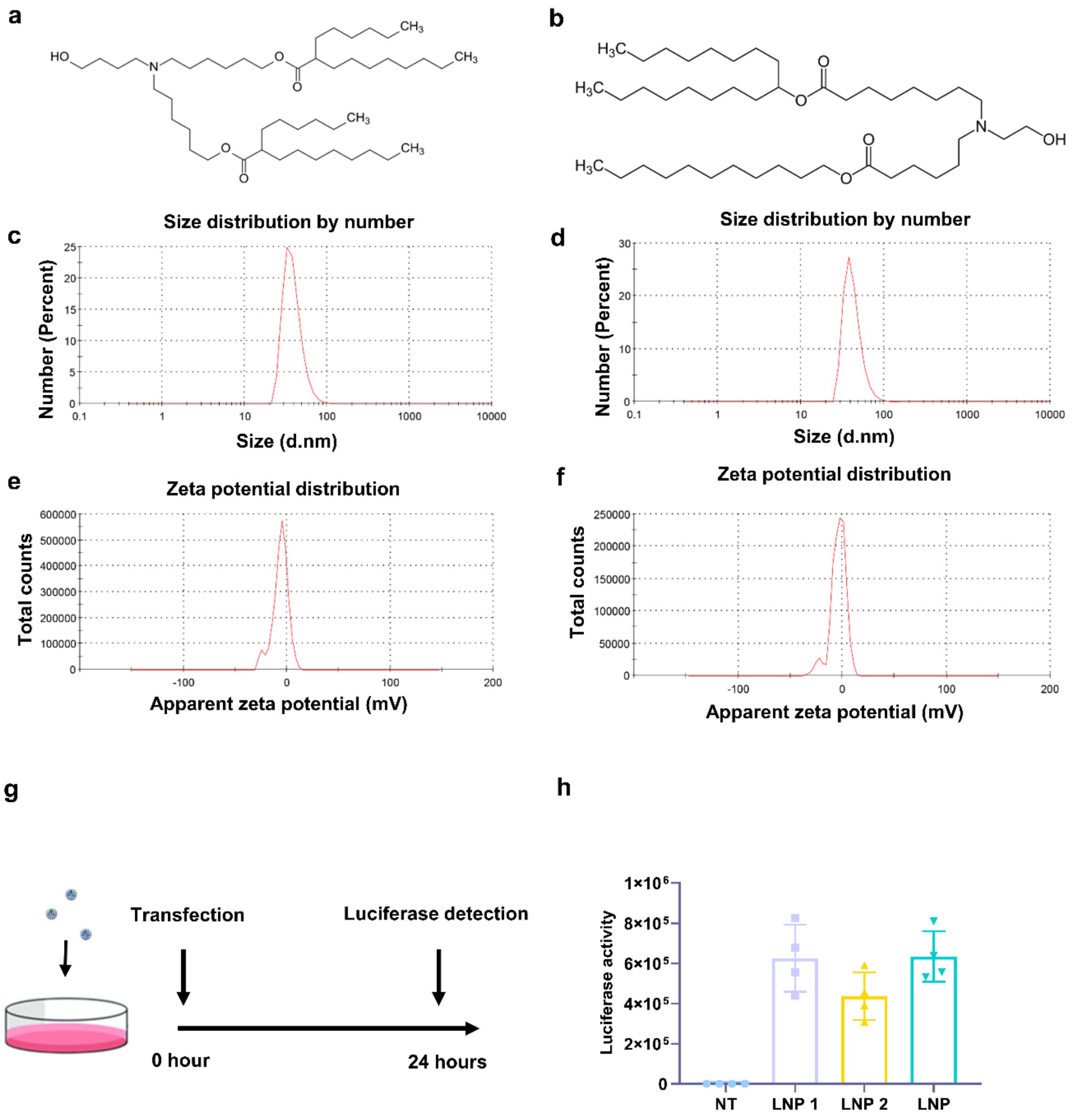

Figure 5 (a-b) The molecular structural formula of the two FDA-approved ionizable lipids (a, LNP 1; b, LNP 2). Particle size distributions of the circRNA-LNP 1 (**c**) and circRNA-LNP 2 (**d**) complex. Zeta potentials of the circRNA-LNP 1 (**e**) and circRNA-LNP 2 (**f**) complex. **(g)** A workflow of the assay in vitro. OVA (257-264)-luciferase-coding circRNA was encapsulated with different LNPs for transfection. 24 hours after transfection, cells were collected for luciferase activity detection assay. **(h)** Luciferase activity detection in HEK293T cells.

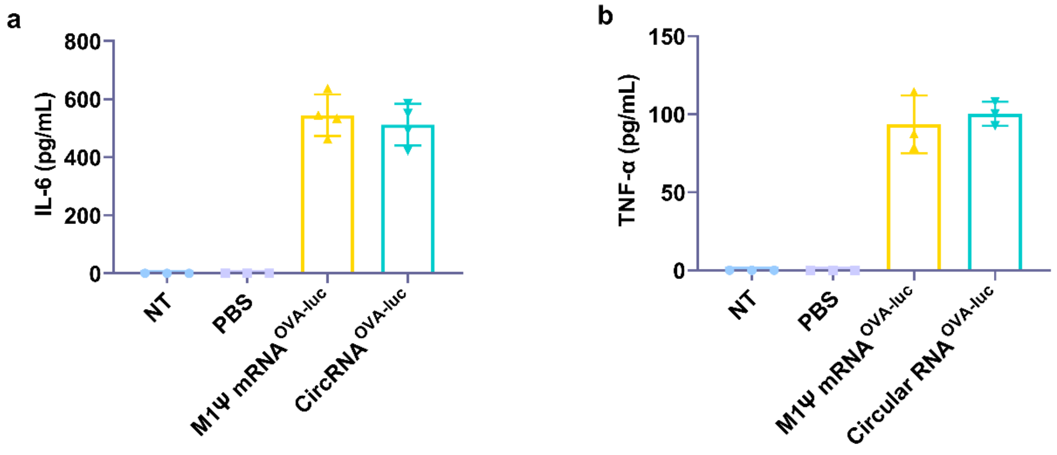

Figure 6 Comparison of serum cytokine release after RNA-LNP administration in vivo. OVA (257-264)-luciferase-coding circRNA (circRNA$^{OVA\text{-}luc}$, 10 μg circRNA per mouse), equimolar amount of M1Ψ mRNA (M1Ψ mRNA$^{OVA\text{-}luc}$) and PBS were encapsuled with LNP and intramuscularly administrated. 24 hours later, Elisa assay was carried out to detect serum (**a**) IL-6 and (**b**) TNF-α secretion.

a

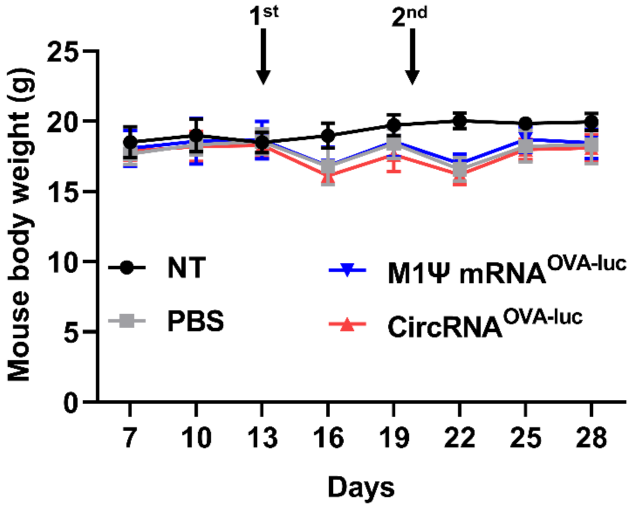

b

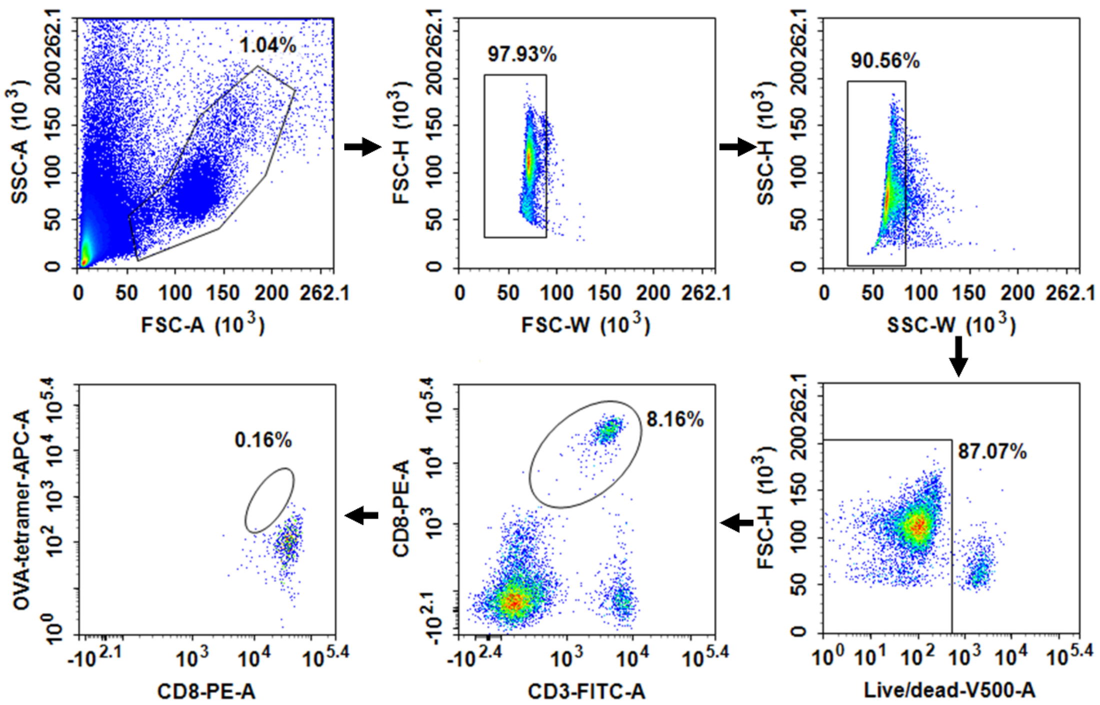

Figure 7 (a) Average body weight of the mice in OVA-MC38 tumor model. **(b)** Gating strategy of the flow cytometry data for anti-OVA 257-264 peptide (SIINFEKL) T cell detection in Figure 4C.

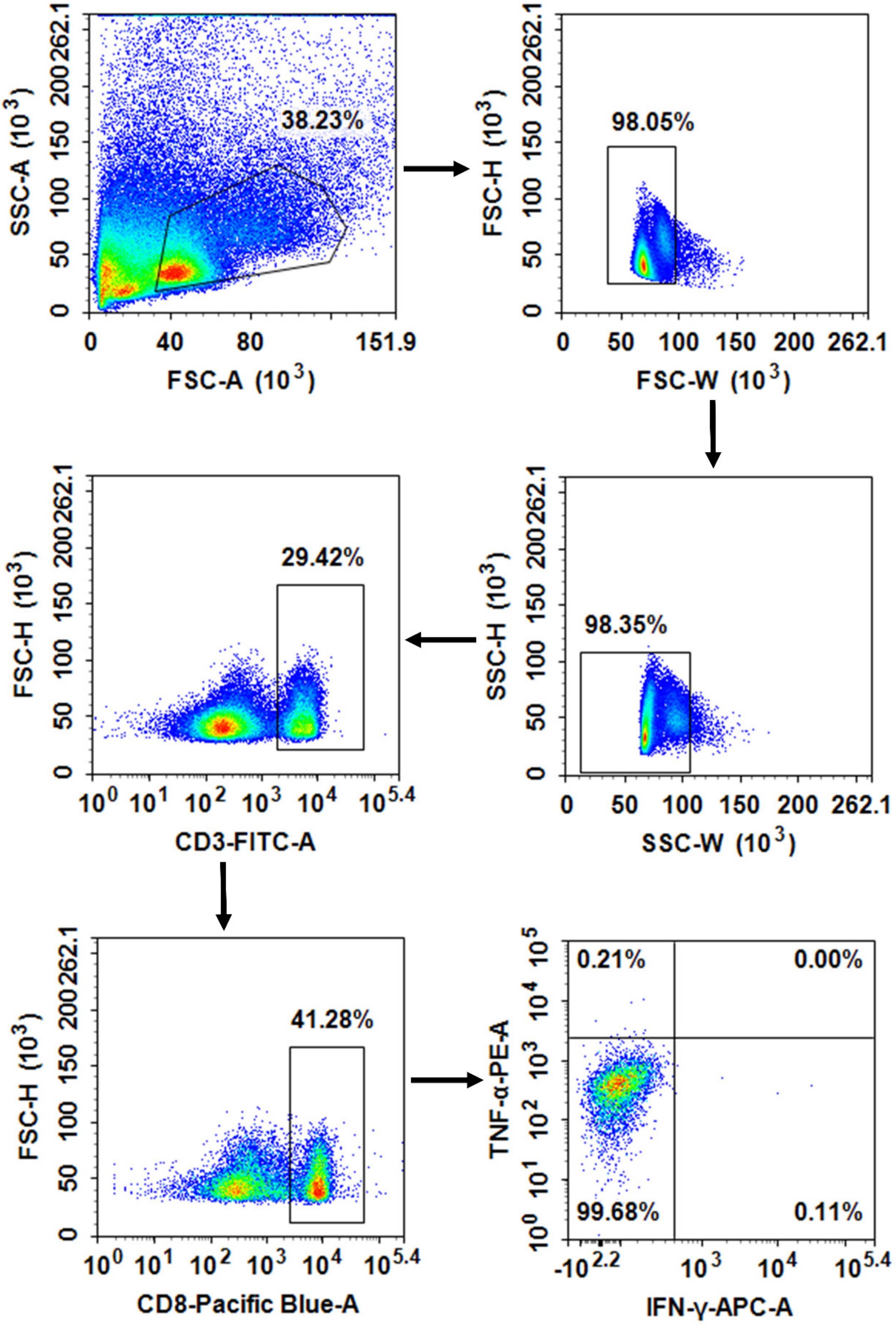

Figure 8 Gating strategy of the flow cytometry data for detecting the percentage of IFN-γ and TNF-α positive $CD8^+$ T cells in Figure 4E.

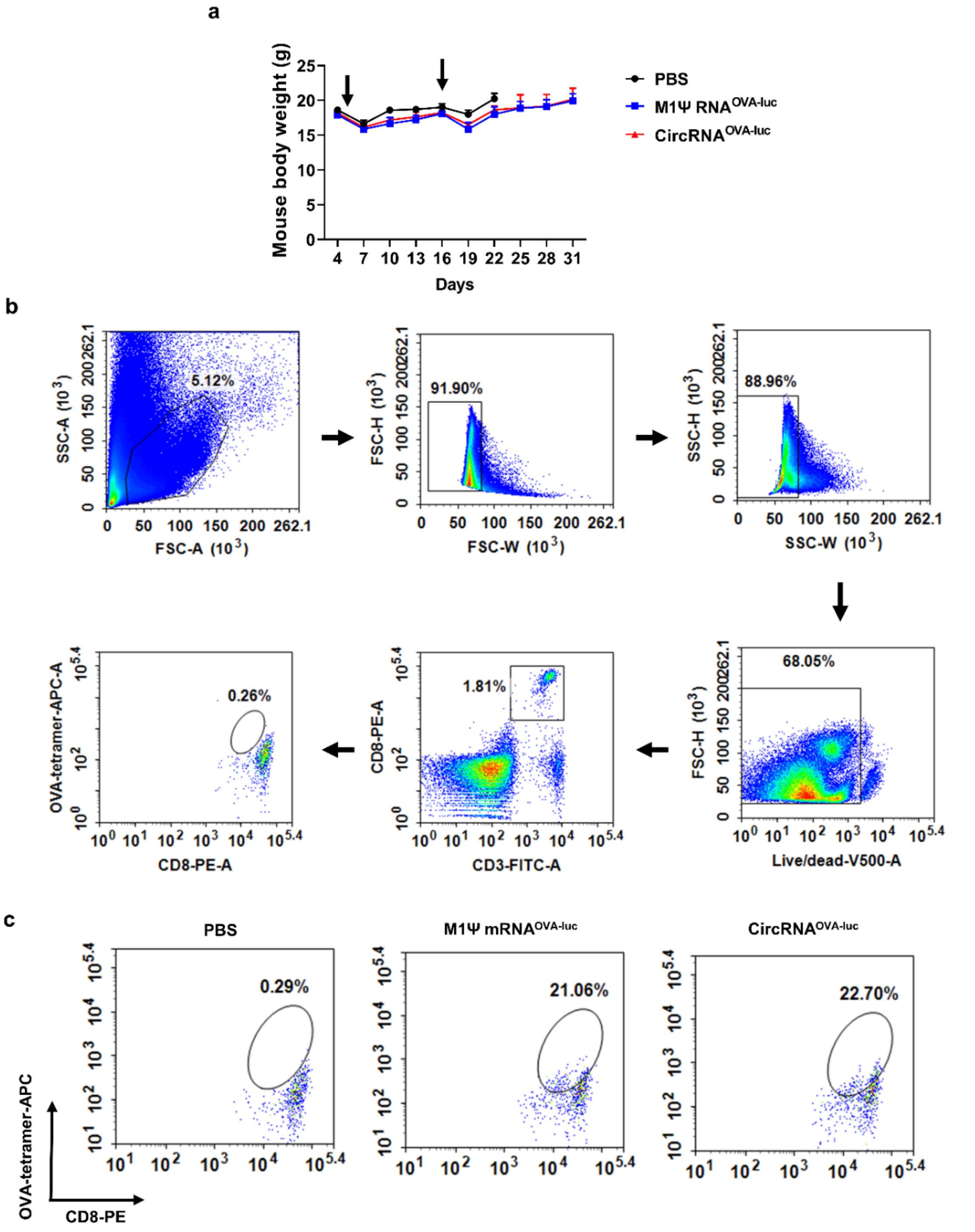

Figure 9 (a) Average body weight of mice during the experiment of Figure 5A. Gating strategy **(b)** and representative flow dot plots **(c)** for anti-OVA 257-264 peptide (SIINFEKL) T cell detection in Figure 5H.

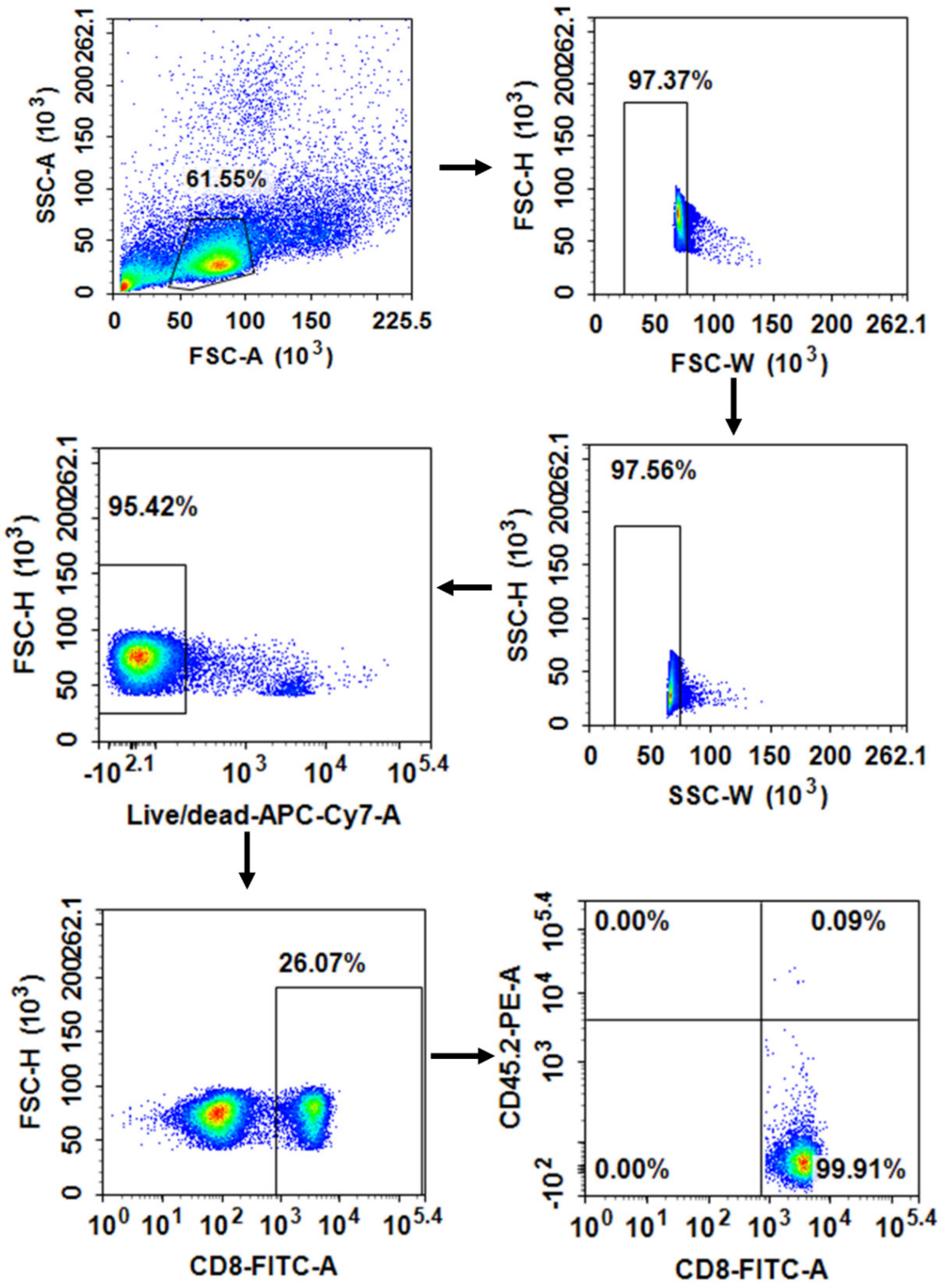

Figure 10 Gating strategy of the flow cytometry data for detecting the OT-I ($CD45.2^+$) T cells in all the $CD8^+$ cells.

Supplementary Table 1

Linear D2GFP RNA templete	taatacgactcactatagggaaataagagagaaaagaagagtaagaagaaatataagagccaccgccaccatggtg<br>agcaagggcgaggagctgttcaccggggtggtgcccatcctggtcgagctggacggcgacgtaaacggccacaa<br>gttcagcgtgtccggcgagggcgagggcgatgccacctacggcaagctgaccctgaagttcatctgcaccaccgg<br>caagctgcccgtgccctggcccaccctcgtgaccaccctgacctacggcgtgcagtgcttcagccgctaccccgac<br>cacatgaagcagcacgacttcttcaagtccgccatgcccgaaggctacgtccaggagcgcaccatcttcttcaagga<br>cgacggcaactacaagacccgcgccgaggtgaagttcgagggcgacaccctggtgaaccgcatcgagctgaagg<br>gcatcgacttcaaggaggacggcaacatcctggggcacaagctggagtacaactacaacagccacaacgtctatat<br>catggccgacaagcagaagaacggcatcaaggtgaacttcaagatccgccacaacatcgaggacggcagcgtgc<br>agctcgccgaccactaccagcagaacacccccatcggcgacggccccgtgctgctgcccgacaaccactacctga<br>gcacccagtccgccctgagcaaagaccccaacgagaagcgcgatcacatggtcctgctggagttcgtgaccgccg<br>ccgggatcactctcggcatggacgagctgtacaagaagcttagccatggcttcccgccggaggtggaggagcagg<br>atgatggcacgctgcccatgtcttgtgcccaggagagcgggatggaccgtcaccctgcagcctgtgcttctgctagg<br>atcaatgtgtaggctgccttctgcggggcttgccttctggccatgcccttcttctctcccttgcacctgtacctcttggtctt<br>tgaataaagcctgagtaggaagt
Circular D2GFP RNA templete	taatacgactcactataggggggagaccctcgaccgtcgattgtccactggtcaacaatagatgacttacaactaatcgg<br>aaggtgcagagactcgacgggagctaccctaacgtcaagacgagggtaaagagagagtccaattctcaaagccaat<br>aggcagtagcgaaagctgcaagagaatgaaaatccgttgaccttaaacggtcgtgtgggttcaagtccctccacccc<br>cacgccggaaacgcaatagccgaaaaacaaaaaacaaaaaaaacaaaaaaaaaaccaaaaaaacaaaacacatct<br>agattaaaacagcctgtgggttgatcccacccacaggcccattgggcgctagcactctggtatcacggtacctttgtgc<br>gcctgttttataccccctcccccaactgtaacttagaagtaacacacaccgatcaacagtcagcgtggcacaccagcc<br>acgttttgatcaagcacttctgttaccccggactgagtatcaatagactgctcacgcggttgaaggagaaagcgttcgtt<br>atccggccaactacttcgaaaaacctagtaacaccgtggaagttgcagagtgtttcgctcagcactaccccagtgtag<br>atcaggtcgatgagtcaccgcattccccacgggcgaccgtggcggtggctgcgttggcggcctgcccatggggaa<br>acccatgggacgctctaatacagacatggtgcgaagagtctattgagctagttggtagtcctccggcccctgaatgcg<br>gctaatcctaactgcggagcacacaccctcaagccagagggcagtgtgtcgtaacgggcaactctgcagcggaacc<br>gactactttgggtgtccgtgtttcattttattcctatactggctgcttatggtgacaattgagagatcgttaccatatagctatt<br>ggattggccatccggtgactaatagagctattatatatccctttgttgggtttataccacttagcttgaaagaggttaaaac<br>attacaattcattgttaagttgaatacagcaaaatggtgagcaagggcgaggagctgttcaccggggtggtgcccatc

	ctggtcgagctggacggcgacgtaaacggccacaagttcagcgtgtccggcgagggcgagggcgatgccaccta<br>cggcaagctgaccctgaagttcatctgcaccaccggcaagctgcccgtgccctggcccaccctcgtgaccaccctg<br>acctacggcgtgcagtgcttcagccgctaccccgaccacatgaagcagcacgacttcttcaagtccgccatgcccga<br>aggctacgtccaggagcgcaccatcttcttcaaggacgacggcaactacaagacccgcgccgaggtgaagttcga<br>gggcgacaccctggtgaaccgcatcgagctgaagggcatcgacttcaaggaggacggcaacatcctggggcaca<br>agctggagtacaactacaacagccacaacgtctatatcatggccgacaagcagaagaacggcatcaaggtgaactt<br>caagatccgccacaacatcgaggacggcagcgtgcagctcgccgaccactaccagcagaacacccccatcggcg<br>acggccccgtgctgctgcccgacaaccactacctgagcacccagtccgccctgagcaaagaccccaacgagaagc<br>gcgatcacatggtcctgctggagttcgtgaccgccgccgggatcactctcggcatggacgagctgtacaagaagctt<br>agccatggcttcccgccggaggtggaggagcaggatgatggcacgctgcccatgtcttgtgcccaggagagcggg<br>atggaccgtcaccctgcagcctgtgcttctgctaggatcaatgtgtaggtcgacaaaaaacaaaaaacaaaacggcta<br>ttatgcgttaccggcgagacgctacggacttaaataattgagccttaaagaagaaattctttaagtggatgctctcaaact<br>cagggaaacctaaatctagttatagacaaggcaatcctgagccaagccgaagtagtaattagtaagaccagtggaca<br>atcgacggataacagcatatctag
Circular<br>OVA<br>(257-264;<br>SIINFEKL)-<br>luciferase<br>RNA<br>templete	taatacgactcactataggggagaccctcgaccgtcgattgtccactggtcaacaatagatgacttacaactaatcgg<br>aaggtgcagagactcgacgggagctaccctaacgtcaagacgagggtaaagagagagtccaattctcaaagccaat<br>aggcagtagcgaaagctgcaagagaatgaaaatccgttgaccttaaacggtcgtgtgggttcaagtccctccacccc<br>cacgccggaaacgcaatagccgaaaaacaaaaaacaaaaaaaacaaaaaaaaaaccaaaaaaacaaaacacatct<br>agattaaaacagcctgtgggttgatcccacccacaggcccattgggcgctagcactctggtatcacggtacctttgtgc<br>gcctgttttatacccccctcccccaactgtaacttagaagtaacacacaccgatcaacagtcagcgtggcacaccagcc<br>acgttttgatcaagcacttctgttaccccggactgagtatcaatagactgctcacgcggttgaaggagaaagcgttcgtt<br>atccggccaactacttcgaaaaacctagtaacaccgtggaagttgcagagtgtttcgctcagcactaccccagtgtag<br>atcaggtcgatgagtcaccgcattccccacgggcgaccgtggcggtggctgcgttggcggcctgcccatggggaa<br>acccatgggacgctctaatacagacatggtgcgaagagtctattgagctagttggtagtcctccggcccctgaatgcg<br>gctaatcctaactgcggagcacacaccctcaagccagagggcagtgtgtcgtaacgggcaactctgcagcggaacc<br>gactactttgggtgtccgtgtttcattttattcctatactggctgcttatggtgacaattgagagatcgttaccatatagctatt<br>ggattggccatccggtgactaatagagctattatatatccctttgttgggtttataccacttagcttgaaagaggttaaaac<br>attacaattcattgttaagttgaatacagcaaaatgcttgagcagcttgagagtataatcaactttgaaaaactgactgaat<br>ggaccagtggggatccaccggtcatggaagacgccaaaaacataaagaaaggcccggcgccattctatccgctgg

	aagatggaaccgctggagagcaactgcataaggctatgaagagatacgccctggttcctggaacaattgcttttacagatgcacatatcgaggtggacatcacttacgctgagtacttcgaaatgtccgttcggttggcagaagctatgaaacgatatgggctgaatacaaatcacagaatcgtcgtatgcagtgaaaactctcttcaattctttatgccggtgttgggcgcgttatttatcggagttgcagttgcgcccgcgaacgacatttataatgaacgtgaattgctcaacagtatgggcatttcgcagcctaccgtggtgttcgtttccaaaaaggggttgcaaaaaattttgaacgtgcaaaaaaagctcccaatcatccaaaaaattattatcatggattctaaaacggattaccagggatttcagtcgatgtacacgttcgtcacatctcatctacctcccggttttaatgaatacgattttgtgccagagtccttcgatagggacaagacaattgcactgatcatgaactcctctggatctactggtctgcctaaaggtgtcgctctgcctcatagaactgcctgcgtgagattctcgcatgccagagatcctatttttggcaatcaaatcattccggatactgcgattttaagtgttgttccattccatcacggttttggaatgtttactacactcggatatttgatatgtggatttcgagtcgtcttaatgtatagatttgaagaagagctgtttctgaggagccttcaggattacaagattcaaagtgcgctgctggtgccaaccctattctccttcttcgccaaaagcactctgattgacaaatacgatttatctaatttacacgaaattgcttctggtggcgctcccctctctaaggaagtcggggaagcggttgccaagaggttccatctgccaggtatcaggcaaggatatgggctcactgagactacatcagctattctgattacacccgagggggatgataaaccgggcgcggtcggtaaagttgttccattttttgaagcgaaggttgtggatctggataccgggaaaacgctgggcgttaatcaaagaggcgaactgtgtgtgagaggtcctatgattatgtccggttatgtaaacaatccggaagcgaccaacgccttgattgacaaggatggatggctacattctggagacatagcttactgggacgaagacgaacacttcttcatcgttgaccgcctgaagtctctgattaagtacaaaggctatcaggtggctcccgctgaattggaatccatcttgctccaacaccccaacatcttcgacgcaggtgtcgcaggtcttcccgacgatgacgccggtgaacttcccgccgccgttgttgttttggagcacggaaagacgatgacggaaaaagagatcgtggattacgtcgccagtcaagtaacaaccgcgaaaaagttgcgcggaggagttgtgtttgtggacgaagtaccgaaaggtcttaccggaaaactcgacgcaagaaaaatcagagagatcctcataaaggccaagaagggcggaaagatcgccgtgtgagtcgacaaaaaacaaaaaacaaaacggctattatgcgttaccggcgagacgctacggacttaaataattgagccttaaagaagaaattctttaagtggatgctctcaaactcagggaaacctaaatctagttatagacaaggcaatcctgagccaagccgaagtagtaattagtaagaccagtggacaatcgacggataacagcatatctag
Linear OVA (257-264; SIINFEKL)-luciferase RNA templete	taatacgactcactatagggaaataagagagaaaagaagagtaagaagaaatataagagccaccgccaccatgcttgagcagcttgagagtataatcaactttgaaaaactgactgaatggaccagtggggatccaccggtcatggaagacgccaaaaacataaagaaaggcccggcgccattctatccgctggaagatggaaccgctggagagcaactgcataaggctatgaagagatacgccctggttcctggaacaattgcttttacagatgcacatatcgaggtggacatcacttacgctgagtacttcgaaatgtccgttcggttggcagaagctatgaaacgatatgggctgaatacaaatcacagaatcgtcgtatgcagtgaaaactctcttcaattctttatgccggtgttgggcgcgttatttatcggagttgcagttgcgcccgcgaacgacatttata

	atgaacgtgaattgctcaacagtatgggcatttcgcagcctaccgtggtgttcgtttccaaaaaggggttgcaaaaaatt<br>ttgaacgtgcaaaaaaagctcccaatcatccaaaaaattattatcatggattctaaaacggattaccagggatttcagtc<br>gatgtacacgttcgtcacatctcatctacctcccggttttaatgaatacgattttgtgccagagtccttcgatagggacaa<br>gacaattgcactgatcatgaactcctctggatctactggtctgcctaaaggtgtcgctctgcctcatagaactgcctgcg<br>tgagattctcgcatgccagagatcctatttttggcaatcaaatcattccggatactgcgattttaagtgttgttccattccat<br>cacggttttggaatgtttactacactcggatatttgatatgtggatttcgagtcgtcttaatgtatagatttgaagaagagct<br>gtttctgaggagccttcaggattacaagattcaaagtgcgctgctggtgccaaccctattctccttcttcgccaaaagca<br>ctctgattgacaaatacgatttatctaatttacacgaaattgcttctggtggcgctcccctctctaaggaagtcggggaag<br>cggttgccaagaggttccatctgccaggtatcaggcaaggatatgggctcactgagactacatcagctattctgattac<br>acccgagggggatgataaaccgggcgcggtcggtaaagttgttccattttttgaagcgaaggttgtggatctggatac<br>cgggaaaacgctgggcgttaatcaaagaggcgaactgtgtgtgagaggtcctatgattatgtccggttatgtaaacaat<br>ccggaagcgaccaacgccttgattgacaaggatggatggctacattctggagacatagcttactgggacgaagacg<br>aacacttcttcatcgttgaccgcctgaagtctctgattaagtacaaaggctatcaggtggctcccgctgaattggaatcc<br>atcttgctccaacaccccaacatcttcgacgcaggtgtcgcaggtcttcccgacgatgacgccggtgaacttcccgcc<br>gccgttgttgttttggagcacggaaagacgatgacggaaaaagagatcgtggattacgtcgccagtcaagtaacaac<br>cgcgaaaaagttgcgcggaggagttgtgtttgtggacgaagtaccgaaaggtcttaccggaaaactcgacgcaaga<br>aaaatcagagagatcctcataaaggccaagaagggcggaaagatcgccgtgtaagctgccttctgcggggcttgcc<br>ttctggccatgcccttcttctctcccttgcacctgtacctcttggtctttgaataaagcctgagtaggaagt
CVB3 IRES templete	ttaaaacagcctgtgggttgatcccacccacaggcccattgggcgctagcactctggtatcacggtacctttgtgcgcc<br>tgttttatacccctcccccaactgtaacttagaagtaacacacaccgatcaacagtcagcgtggcacaccagccacgt<br>tttgatcaagcacttctgttaccccggactgagtatcaatagactgctcacgcggttgaaggagaaagcgttcgttatcc<br>ggccaactacttcgaaaaacctagtaacaccgtggaagttgcagagtgtttcgctcagcactaccccagtgtagatca<br>ggtcgatgagtcaccgcattccccacgggcgaccgtggcggtggctgcgttggcggcctgcccatggggaaaccc<br>atgggacgctctaatacagacatggtgcgaagagtctattgagctagttggtagtcctccggcccctgaatgcggcta<br>atcctaactgcggagcacacaccctcaagccagagggcagtgtgtcgtaacgggcaactctgcagcggaaccgact<br>actttgggtgtccgtgtttcattttattcctatactggctgcttatggtgacaattgagagatcgttaccatatagctattgga<br>ttggccatccggtgactaatagagctattatatatccctttgttgggtttataccacttagcttgaaagaggttaaaacatta<br>caattcattgttaagttgaatacagcaaa
EV33 IRES	ttaaaacagcctgtgggttgatcccacccacagggcccattgggcgctagcactctggtatcacggtacccttgtgcg

templete	cctgttttatgtcccttccctcaactgtaacttagaagtaacgcacaccgatcaacagtcagcgtggcacaccagccatgttttgatcaagcacttctgttaccccggaccgagtatcaacagactgctcacgcggttgaaggagaaagtgttcgttatccggccaactacttcgaaaaacctagtaacaccatggaagttgcagagtgtttcgctcagcactaccccagtgtagatcaggtcgatgagtcaccgcatccccccacgggcgaccgtggcggtggctgcgttggcggcctgcctatgggggaacccataggacgctctaatacagacatggtgcgaagagtccattgagctagttggtagtcctccggcccctgaatgcggctaatcctaactgcggagcacacaccttcaagccagagggcagtgtgtcgtaacgggcaactctgcagcggaaccgactactttgggtgtccgtgtttcattttattcttatactggctgcttatggtgacaattgagagattgttaccatatagctattggattggccatccagtgactagcagagctattatatacctctttgttgggtttataccacctaatttgaaagaagttaaaacattagaattcattattaaattgaataca
EV29 IRES templete	ttaaaacagcctgtgggttgatcccacccacagggcccactgggcgctagcactctggtatcacggtacctttgtgcgcctgttttatacttcctcccccaactgcaacttagaagtaacacaaaccgatcaacagtcagcgtggcacaccagccacgttttgatcaaacacttctgttaccccggactgagtatcaatagactgctcacgcggttgaaggagaaaacgttcgttatccggccaactacttcgagaaacctagtaacgccatggaagttgtggagtgtttcgctcagcactaccccagtgtagatcaggttgatgagtcaccgcattccccacgggtgaccgtggcggtggctgcgttggcggcctgcccatggggaaacccatgggacgctcttatacagacatggtgcgaagagtctattgagctagttggtagtcctccggcccctgaatgcggctaatcccaactgcggagcatacactctcaagccagagggtagtgtgtcgtaatgggcaactctgcagcggaaccgactactttgggtgtccgtgtttcattttattcctatactggctgcttatggtgacaattgagagattgttaccatatagctattggattggccatccggtgactaacagagctattatatatctttttgttgggtttataccacttagcttgaaagaggttaaaactctacattacattttaatactgaacaccgcaaa
VCIP IRES templete	gacctcgtgaaataaaagtgcagaaaacaaacccaggcgatcacagcagcagccgccgcggcagcagcaccaacagcaggaggagcaggaggagccggaggaggaggaggaggaggaggcaaagttagagttggggctggcgctccggagttgctgggctcagcgcagctcccattcattaaggaaccagctgcggaggaaggtggccgagcgcccgcgctgcccactcgctcgctcgcgcactcagacgcgcgccacaacagcgcgccccaagctgcgcagctctgcaaaagtttctgctcgggatctggctctcttccccttggactttagaacgatttagggttgacagaggaaagcagaggcgcgcaggaggagcagaaaacaccaccttctgcagttggaggcaggcagccccggctgcactctagccgccgcgcccggagccggggccgacccgccactatccgcagcagcctcggccaggaggcgacccgggcgcctgggtgtgtggctgctgttgcgggacgtcttcgcggggcgggaggctcgcgccgcagccagcgcc
T7 promoter	TAATACGACTCACTATAGGG
5'UTR	AAATAAGAGAGAAAAGAAGAGTAAGAAGAAATATAAGAGCCACC

3'UTR	GCTGCCTTCTGCGGGGCTTGCCTTCTGGCCATGCCCTTCTTCTCTCCCTT<br>GCACCTGTACCTCTTGGTCTTTGAATAAAGCCTGAGTAGGAAGT
